# TGF-β inhibition combined with cytotoxic nanomedicine normalizes triple negative breast cancer microenvironment towards anti-tumor immunity

**DOI:** 10.7150/thno.36936

**Published:** 2020-01-12

**Authors:** Myrofora Panagi, Chrysovalantis Voutouri, Fotios Mpekris, Panagiotis Papageorgis, Margaret R Martin, John D Martin, Philippos Demetriou, Chryso Pierides, Christiana Polydorou, Andreas Stylianou, Maria Louca, Laura Koumas, Paul Costeas, Kazunori Kataoka, Horacio Cabral, Triantafyllos Stylianopoulos

**Affiliations:** 1 Cancer Biophysics Laboratory, Department of Mechanical and Manufacturing Engineering, University of Cyprus, Nicosia, Cyprus.; 2 Department of Life Sciences, Program in Biological Sciences, European University Cyprus, Nicosia, Cyprus.; 3 Department of Bioengineering, Graduate School of Engineering, The University of Tokyo, Bunkyo, Tokyo, Japan.; 4 The Center for the Study of Haematological Malignancies, Nicosia, Cyprus.; 5 Karaiskakio Foundation, Nicosia, Cyprus.; 6 Innovation Center of NanoMedicine, Kawasaki Institute of Industrial Promotion, Kawasaki, Kanagawa, Japan.; 7 Institute of Future Initiatives, The University of Tokyo, Bunkyo, Tokyo, Japan.

**Keywords:** tumor microenvironment, vascular perfusion, normalization, nanomedicine, immunostimulation, immunotherapy

## Abstract

Tumor normalization strategies aim to improve tumor blood vessel functionality (i.e., perfusion) by reducing the hyper-permeability of tumor vessels or restoring compressed vessels. Despite progress in strategies to normalize the tumor microenvironment (TME), their combinatorial antitumor effects with nanomedicine and immunotherapy remain unexplored.

**Methods**: Here, we re-purposed the TGF-β inhibitor tranilast, an approved anti-fibrotic and antihistamine drug, and combined it with Doxil nanomedicine to normalize the TME, increase perfusion and oxygenation, and enhance anti-tumor immunity. Specifically, we employed two triple-negative breast cancer (TNBC) mouse models to primarily evaluate the therapeutic and normalization effects of tranilast combined with doxorubicin and Doxil. We demonstrated the optimized normalization effects of tranilast combined with Doxil and extended our analysis to investigate the effect of TME normalization to the efficacy of immune checkpoint inhibitors.

**Results**: Combination of tranilast with Doxil caused a pronounced reduction in extracellular matrix components and an increase in the intratumoral vessel diameter and pericyte coverage, indicators of TME normalization. These modifications resulted in a significant increase in tumor perfusion and oxygenation and enhanced treatment efficacy as indicated by the notable reduction in tumor size. Tranilast further normalized the immune TME by restoring the infiltration of T cells and increasing the fraction of T cells that migrate away from immunosuppressive cancer-associated fibroblasts. Furthermore, we found that combining tranilast with Doxil nanomedicine, significantly improved immunostimulatory M1 macrophage content in the tumorigenic tissue and improved the efficacy of the immune checkpoint blocking antibodies anti-PD-1/anti-CTLA-4.

**Conclusion**: Combinatorial treatment of tranilast with Doxil optimizes TME normalization, improves immunostimulation and enhances the efficacy of immunotherapy.

## Introduction

Advances in cancer nanomedicine have led to the development of several new systemically administered nanoparticles to treat various tumor types, including breast and pancreatic cancers 1. The enhanced permeability and retention (EPR) effect has served as a key rationale for using nanoparticles to target cancer cells or improve their pharmacokinetic properties for the treatment of solid tumors. As a result, clinically approved nanomedicines have succeeded in reducing adverse effects - owing to the EPR effect and preferential accumulation to the tumor - but survival benefits are modest in most cases 1, 2.

Indeed, the efficacy of nanomedicines to solid tumors is hindered by abnormalities in the structure of the tumor vasculature, which can reduce drastically tumor perfusion and as a result the systemic delivery of any blood-borne therapeutic agent 3, 4. In many tumor types, blood vessels are hyper-permeable, forming large interendothelial openings, which is an effect of increased expression of pro-angiogenic factors (e.g. vascular endothelial growth factor, VEGF) that drive tumor-induced angiogenesis 5, 6. Vessel hyper-permeability causes fluid loss from the vascular to the interstitial space of the tumor, reducing tumor blood flow (i.e., perfusion) 7. Additionally, the dense tumor extracellular matrix and the rapid proliferation of cancer cells in the confined space surrounding the tumor result in the development of intratumoral mechanical forces that can strain components of the TME, including blood vessels, thus causing vessel compression 8-10. Both vessel hyper-permeability and compression can reduce tumor blood flow, rendering tumors hypo-perfused and hypoxic. Impaired blood supply and hypoxia hinder nanoparticle delivery but also help cancer cells evade the immune system and increase their invasive and metastatic potential 11-13. Particularly, hypo-perfusion can reduce the number of immune cells that can infiltrate into the tumor, while hypoxia renders TME immunosuppressive and attenuates the killing potential of effector immune cells 13-16.

Vascular normalization and normalization of the tumor stroma are two strategies that have been clinically used separately, to improve perfusion and efficacy of chemotherapy 7, 17, 18. Vascular normalization is based on the judicious use of anti-angiogenic agents to target vessel permeability 6, 19, while normalization of the tumor interstitial space is based on the use of drugs exerting anti-fibrotic properties that normalize the tumor extracellular matrix in order to alleviate forces and decompress tumor vessels 8, 20-23. There are also agents that can target both tumor blood vessel permeability and interstitial space components, inducing TME normalization 24. These normalization strategies have been clinically applied and found to improve treatment efficacy in a variety of solid tumors 3, 25. It has also been suggested that these strategies can improve immunostimulation and thus, enhance cancer cell killing by immune cells 26-30. Importantly, TME normalization was recently found to improve the efficacy of immune checkpoint blockers in mice with primary as well as metastatic breast cancer 31, 32.

As far as the use of normalization strategies to improve nanomedicine delivery is concerned, vascular normalization has been shown in preclinical models of breast cancer to enhance nanoparticle delivery in a size-dependent manner, improving delivery of particles as large as 40 nm 33, 34. Normalization of the tumor interstitial space has been shown to improve delivery of nanoparticles as large as 125 nm and to reduce growth rates in murine breast tumor models treated with common nanomedicines, such as Abraxane and Doxil 20, 22, 35, 36.

However, there is little evidence that combinatorial use of a normalization agent and nanomedicine can improve TME normalization, enhance antitumor immune responses and improve overall survival 37. The recent development of micellar particles loaded with valsartan, an angiotensin receptor blocker with TME normalization properties, is an example of such an approach 31. To this end, we employed two syngeneic triple negative murine breast tumor models (4T1 and E0771) and used the transforming growth factor (TGF)-β inhibitor tranilast (Rizaben), a clinically approved antihistamine and anti-fibrotic drug, as the normalization agent alone or in combination with doxorubicin or its nanoparticle formulation Doxil (~100 nm, PEGylated liposomal doxorubicin). In previous work, we found that tranilast suppresses the expression of TGF-β target genes involved in collagen (e.g., COL1A1 and COL3A1) and hyaluronan synthesis (e.g., HAS2 and HAS3), as well as, other components of the extracellular matrix (ECM) such as connective tissue growth factor (CTGF) and lysyl oxidase (LOX) gene expression in breast tumors 36. The aim of the study was to investigate whether the normalization effects of tranilst can be optimized with use of low doses of a cytotoxic drug (i.e., doses that cannot lead to primary tumor regression). We demonstrate that combination of tranilast with Doxil nanomedicine, significantly improved blood vessel functionality and oxygenation and delayed tumor growth compared to the other treatments employed. Furthermore, the tranilast-Doxil treatment favored the accumulation of M1-like tumor associated macrophages (TAMs), presumably due to the increase in tumor oxygenation. Additionally, we measured a decrease in the distances between cancer-associated fibroblasts (CAFs) and CD3^+^ T cells. This change in T cell motility towards CAFs indicates that there is less collagen barrier favoring a normal phenotype that is not immune suppressive. Based on these data, we conducted a second study using the 4T1 tumor model to evaluate the therapeutic potential of immune checkpoint blockade under normalized TME conditions. Again, we observed that the combinatorial tranilast-Doxil treatment significantly improved the efficacy of the immune checkpoint blocking anti-CTLA-4 and anti-PD-1 cocktail. Our findings strongly suggest that the effect of TGF-β inhibition is drastically increased when combined with cytotoxic nanomedicine or immunotherapy, proposing a new treatment strategy.

## Methods

A detailed description of all materials and methods employed in the study are presented in the Supporting Information. Here, we provide a summary of them.

**Animal tumor models and treatment protocols.** 4T1 and E0771 tumor models were generated by orthotopic implantation of either 5×10^4^ 4T1 or 1x10^5^ E0771 mouse breast cancer cells in 40 µl of serum-free medium into the mammary fat pad of 6-week old BALB/c and C57BL/6 female mice, respectively. Animals were treated with saline (Control), tranilast (200mg/kg, orally daily 4 days post implantantation), doxorubicin (5mg/kg, intraperitoneally every 3 days after tumors reached an average size of 250mm^3^), Doxil (3mg/kg, intravenously weekly after tumors reached an average size of 250mm^3^) or tranilast-doxorubicin and tranilast-Doxil until time of death or time to reach a maximum tumor burden of 1200mm^3^ 20, 36. Tranilast is a hydrophobic drug and it was dissolved in H_2_O containing 10% NaHCO_3_ at 70°C. Animal survival was monitored daily from day 4 post implantation until completion of experiment, while tumor size was measured every 2-3 days. Alterations within the TME were studied by performing a second experiment whereby the same conditions were applied. Prior to sacrifice and tumor excision, animals were anesthetized via Avertin (200mg/kg, intraperitoneal) and intracardially slowly injected with biotinylated lycopersicon esculentum lectin (4mg/kg) and/or pimonidazole HCl (60mg/kg, intraperitoneal) and interstitial fluid pressure was measured using the wick-in-needle method 20, 22, 36.

The effect of immunotherapy on tumor growth was evaluated in a syngeneic 4T1 and E00771 tumor models. Immunotherapy was administrated as a cocktail of 10mg/kg anti-PD-1 (CD279, clone RMP1-14, BioXCell) and 5mg/kg anti-CTLA-4 (CD152, clone 9D9) following dilution in the recommended *InVivo*Pure pH 7.0 Dilution Buffer (BioXCell). The immunotherapy cocktail was administered i.p. on days 14, 17 and 20 post-implantation. Animals were treated with saline, tranilast and Doxil as previously in combination with a non-targeting isotype control antibody (BioXCell).

**Biomechanical analysis.** Characterization of the mechanical properties and calculation of the elastic modulus were determined using an unconfined compression experimental protocol as previously described 10, 38. Growth-induced solid stress was measured using the tumor opening technique as previously described 8, 9, 38. Briefly, after tumor excision, a cut was made throughout the tumor along its longest axis up to 80% of its thickness. The cut caused release of the stresses and the tumor relaxed in a measurable way. The relaxation mode was described by swelling of the interior of the tumor and opening at the periphery (Figure S6).

**Immunohistochemical assessment of tumor microenvironment.** For fluorescent immunohistochemistry and vessel perfusion histology experiments, tumors and lungs were excised, fixed with 4% PFA and embedded in optimal temperature compound (OCT) to produce transverse 20μm-thick and 40μm-thick cryosections of primary tumor and lung tissue, respectively. ECM content was studied using immunostaining against collagen I and hyaluronan, while vascular perfusion was examined following staining against the CD31 endothelial marker and biotinylated tomato lectin. Hypoxic regions within primary tumor and metastatic nodules were detected using the mouse anti-pimonidazole RED 549 conjugate antibody. Pericyte coverage of functional vessels was determined as the ratio of lectin, αSMA and CD31 positive staining to total CD31 area fraction. Macrophage status of 4T1 and E0771 TME was determined following immunostaining with anti-CD11c (HL3, BD Pharmingen 1:100), anti-CD206 (MR5D3, BIO-RAD 1:50) and rat anti-F4/80 (A3-1, BIO-RAD 1:50) antibodies to detect M1-like TAMs, M2-like TAMs and total TAM population, respectively. Quantification of M1-like TAMs was defined as the ratio of CD11c positive signal to F4/80 area fraction. M2-like TAM content was indicated by CD206 area fraction after subtraction of the CD11c and CD206 overlapping signal. All measurements were normalized to DAPI positive staining. The distance between CD3^+^ T cells and αSMA^+^ CAFs was measured using a custom MATLAB script (details in the Supporting Information).

**Flow cytometry.** On day 25 of treatment 4T1 breast tumors were harvested in 1x PBS, minced to fine fragments and incubated with Accumax (Millipore) for 1 hour at room temperature on an end-over-end shaker. Enzymatic digestion was ceased by the addition of RPMI media containing 10% FBS and 1% antibiotic /antimycotic solution. The resulting tissue homogenates were filtered through 70μm cell strainers and single cells suspensions were collected and counted. Cell suspensions were then incubated with fixable viability dye (Invitrogen) for gating of viable cells. Non-specific antibody binding was blocked following incubation with the rat anti-mouse CD16/CD32 mAb (BD Bioscience) for 10min at room temperature. 1x10^6^ cells per sample were labeled with the various fluorochrome conjugated antibodies, washed and resuspended in 1%BSA, 1xPBS buffer. The anti-mouse antibodies used in the experiment are the following; CD4-AF700 (GK1.5, BioLegend), CD127-APC (A7R34, BioLegend), CD206-PE-Cy7 (C068C2, BioLegend), F4/80-APC (BM8, BioLegend), IgG2a-APC (BioLegend), IgG2b-AF488 (BioLegend), IgG2a-PE-Cy7 (BioLegend), Cd11b-eV450 (eBioscience), Gr1-PE (RBG865, BioLegend), CD8a (53-6.7, eBioscience), CD11c-AF700 (HL3, BD Bioscience), Foxp3-AF488 (MF23, BD Bioscience), CD45-V500 (30-F11, BD Bioscience), CD25-PE-Cy7 (PC61.5, BD Bioscience), CD3 (145-2C11, BD Bioscience). Flow cytometry data were obtained using BD FACSAria™ III flow cytometer and analysed using FlowJoX software. Data presented is representative of singlets, live cells.

**Statistical Analysis.** Data are presented as means with standard errors. Groups were compared using Student's *t*-test to study statistical significance. Statistical analyses were performed by comparing the treated groups with the control * and the tranilast-Doxil group with all other treatment groups **. A *P value* of less than or equal to 0.05 was considered statistically significant.

## Results and Discussion

### TME normalization improves the efficacy of both chemo- and nanomedicine

**Figure 1:**
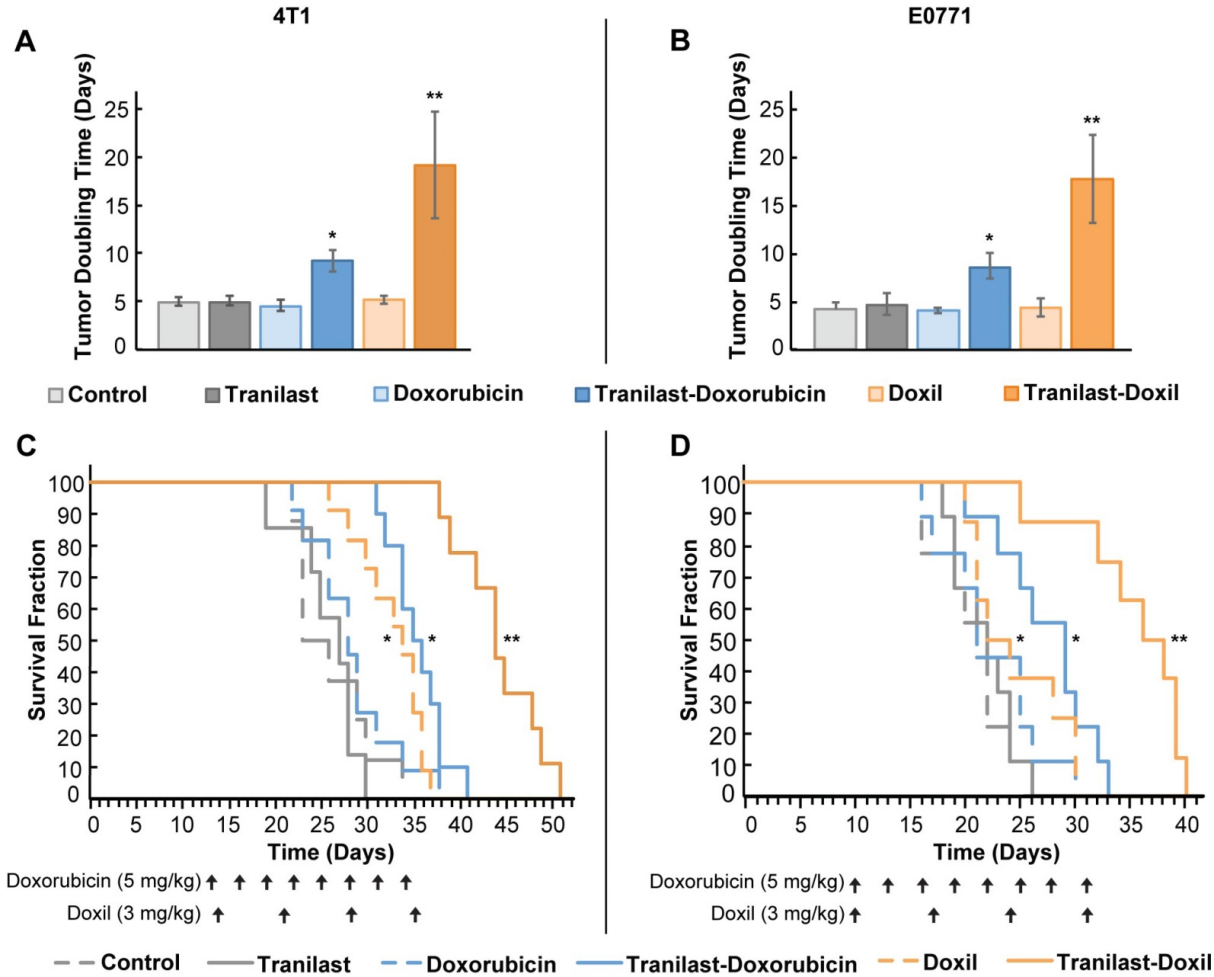
TME normalization increases the efficacy of both chemo- and nanotherapy. Quantification of tumor growth rate, based on the time to reach double the initial volume, for orthotopic 4T1 (**A**) and E0771 (**B**) murine breast tumors implanted in female BALB/c and C57BL/6 mice, respectively. Mice were treated with Control (saline), tranilast (200mg/kg), doxorubicin (5mg/kg), Doxil (3mg/kg), tranilast-doxorubicin and tranilast-Doxil. Tumor volume was measured every 2 days until time of death or time to reach a tumor burden of 1200 mm^3^. Kaplan-Meier survival curves for 4T1 (**C**) and E0771 (**D**) tumor models treated as indicated (arrows). Statistical analyses were performed by comparing the treated groups with the control * and the tranilast-Doxil groups with all other treatment groups **, p≤0.05 (n=8-10).

We re-purposed the clinically approved anti-fibrotic drug tranilast, as the normalization agent, administered in combination with doxorubicin chemotherapy or Doxil nanomedicine. The antitumor efficacy of the combinatorial therapy was evaluated using two orthotopic syngeneic mammary tumor models, 4T1 and E0771, which we have used previously for studying the efficacy of both chemotherapy and nanomedicine 20, 22, 33. Animals were treated with saline (Control), tranilast (200mg/kg, orally), doxorubicin (5mg/kg, intraperitoneal), Doxil (3mg/kg, intravenously) or tranilast-doxorubicin and tranilast-Doxil until the time of physical death or the time required to reach a maximum tumor burden of 1200mm^3^ 20. Mean of administration and dosage of the drugs was based on published pertinent research 20, 36. We found that tranilast, doxorubicin or Doxil monotherapy did not induce any significant delay in tumor growth compared to the untreated group, as indicated by the tumor-doubling time in both tumor models. This confirmed our aim to administer low doses of the two drugs. In contrast, combination of tranilast with doxorubicin caused a 2-fold increase in doubling time of both 4T1 and E0771 tumors, whereas tranilast-Doxil combination produced a more than 3-fold increase in doubling time (Figure 1A, B, Figure S1). Furthermore, tranilast and doxorubicin alone had no effect on animal survival, whereas overall survival was modestly improved after Doxil monotherapy and tranilast-doxorubicin combinatorial therapy compared to controls. Importantly, the survival benefit was significantly improved following tranilast-Doxil combinatorial treatment compared to the rest of the groups (Figure 1C, D). These data demonstrate that the effect of tranilast is necessary for chemotherapy and nanomedicine to exert their anticancer effects and prolong overall survival.

### Doxil nanomedicine enhances tranilast-mediated normalization effects in the primary tumors

**Figure 2:**
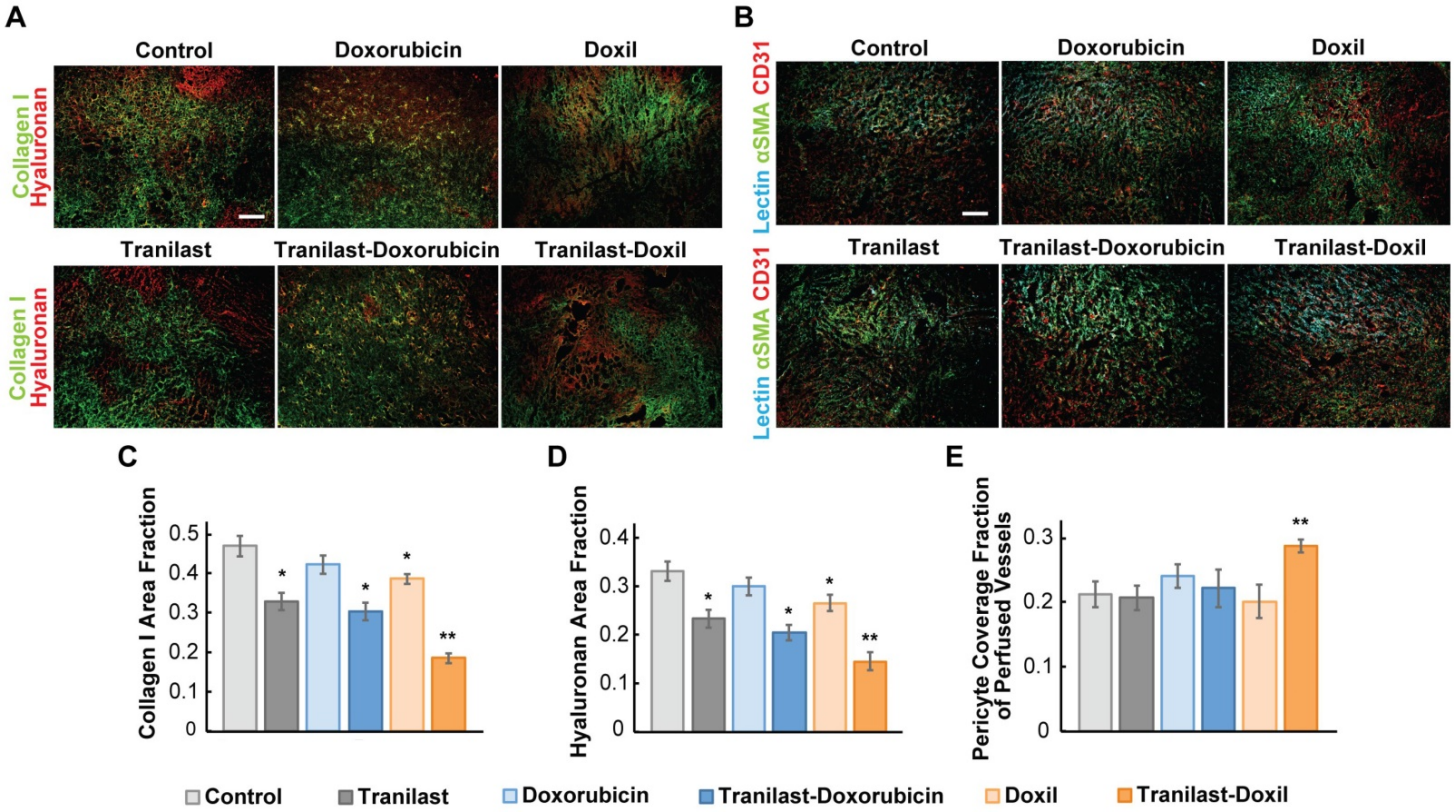
Nanomedicine enhances tranilast-mediated normalization effects in the primary tumors. (**A**) Representative fluorescence images of Collagen I (green) and Hyaluronan (red) immunostaining of 4T1 breast tumors treated as indicated. (**B**) Representative fluorescence images of biotinylated tomato lectin (cyan), CD31 endothelial marker (red) and αSMA pericyte marker (green) immunostaining of 4T1 breast tumors treated as indicated. Quantification of Collagen I (**C**) and Hyaluronan (**D**) area fractions. (**E**) Pericyte coverage of perfused vessels was determined by the co-localization of CD31 and lectin positive staining with αSMA. Statistical analyses were performed by comparing the treated groups with the control * and the tranilast-Doxil group with all other treatment groups **, p≤0.05, (n=8-10). Scale bar: 200μm.

Tranilast has been previously found to reduce mechanical forces and stiffness of breast tumors via reduction of collagen and hyaluronan levels, both being abundantly expressed in such tumors 36. To examine if the significant delay in tumor growth for the tranilast-Doxil group (Figure 1) was initiated by a more efficient normalization of the TME, we performed immunofluorescence staining of tumor cryosections followed by area fraction quantification of these two major extracellular matrix (ECM) components. In line with our previous results, we observed that tranilast alone or in combination with doxorubicin or Doxil monotherapy could decrease the collagen and hyaluronan content of both 4T1 and E0771 tumors, compared to control. Nevertheless, when tranilast was administered with Doxil, collagen and hyaluronan levels were further significantly decreased in both tumor models (Figure 2A, C-D, Figure S2A-C). Our findings suggest that Doxil enhances the interstitial space normalization effects of tranilast and given the known anti-fibrotic properties of doxorubicin 39, this result is likely attributed to the fact that tranilast enhances drug delivery to the primary tumor site (Figure S3) and acts with Doxil to further reduce ECM.

Since ECM constituents develop reciprocal interactions with tumor blood vessels, we next investigated the impact of the combinatorial treatment on blood vessel pericyte coverage, which is a measure of vascular normalization. In normal vessels, pericytes are attached to the vessel wall, sealing the vessel and maintaining vessel permeability to normal values. In tumors, however, pericyte coverage is poor thus contributing to increased vessel permeability. Again, we used immunofluorescence image analysis of cryosections counterstained with the CD31 endothelial marker and the αSMA marker, which when overlapped depicts the presence of pericytes attached to the tumor vasculature. Interestingly, we found that only tranilast-Doxil treated animals exhibited a modest but significant increase in pericyte coverage in 4T1 tumor vessels (Figure 2B, E), thereby highlighting the ability of tranilast-Doxil combination to promote vascular normalization.

### Enhanced tumor normalization by Doxil nanomedicine alleviates intratumoral fluid and solid pressure, increasing perfusion and enhancing tissue oxygenation

**Figure 3:**
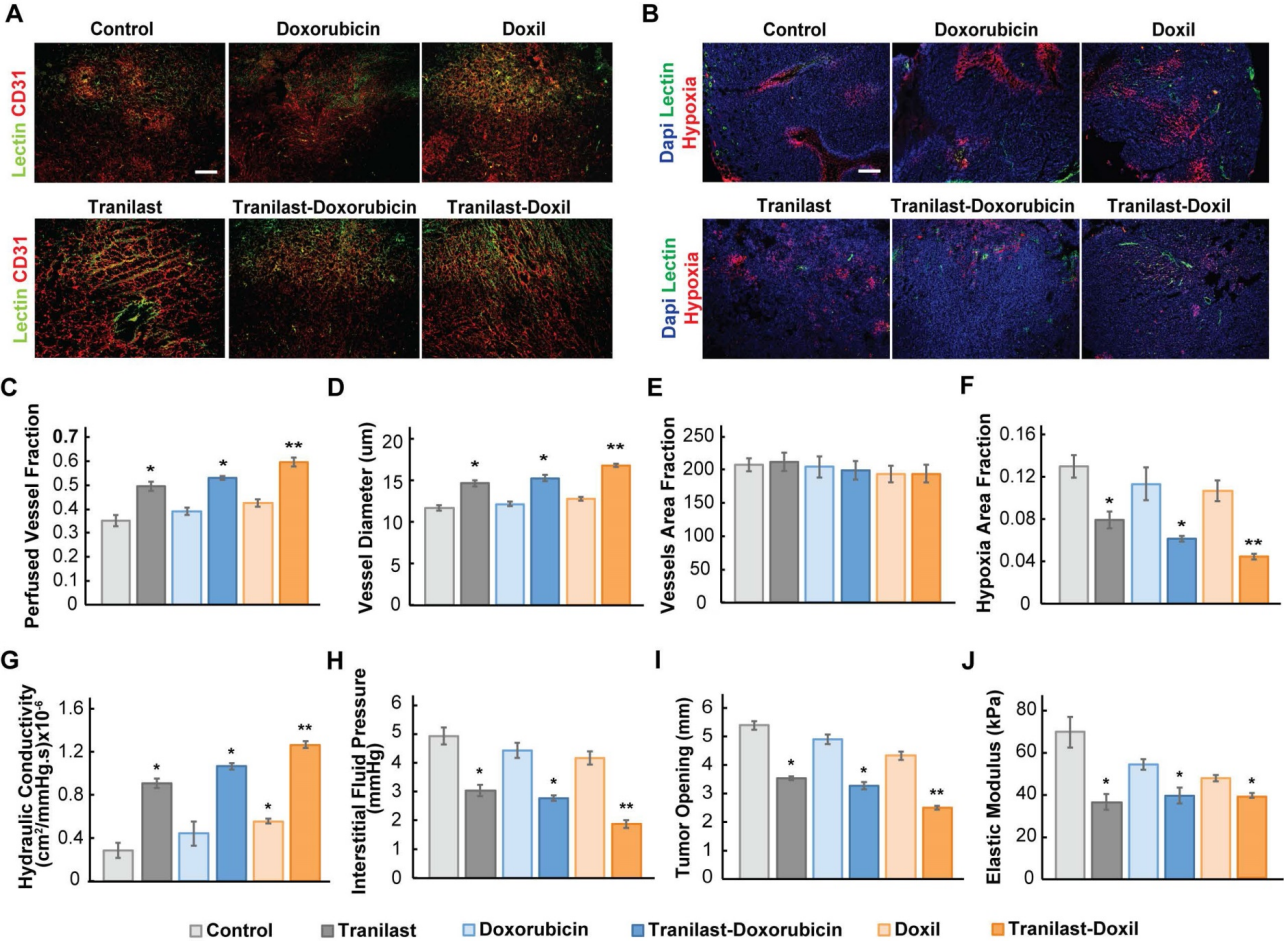
Enhanced tumor normalization by Doxil nanomedicine alleviates intratumoral fluid and solid pressure, increasing perfusion and enhancing tissue oxygenation. (**A**) Representative fluorescence images of 4T1 breast tumor slices immunostained for biotynilated tomato lectin (green) and CD31 (red) after various treatments as indicated. (**B**) Representative fluorescence images of biotynilated tomato lectin (green), pimonidazole (hypoxia, red) immunostaining and DAPI (blue) nuclear staining. (**C**) Quantification of CD31 and lectin co-expression indicating vascular perfusion. (**D**) Quantification of CD31 (red) positive staining indicating mean vessel diameter and (**E**) total blood vessel fraction. (**F**) Hypoxic fraction in E0771 tumors measured following pimonidazole (60 mg/kg) injection and staining. (**G**) Quantification of hydraulic conductivity and (**H**) interstitial fluid pressure measurements of 4T1 tumors treated as indicated. (**I**) Sample-blind measurements of tumor opening and (**J**) elastic modulus. Statistical analyses were performed by comparing the treated groups with the control * and the tranilast-Doxil group with all other treatment groups **, p≤0.05, (n=8-10). Scale bar: 200μm.

To further explore the potential of the enhanced normalization effects caused by the tranilast-Doxil combinatorial treatment strategy on tumor vascular functionality, we measured the percentage of perfused vessels by performing immunofluorescence analysis using 4T1 and E0771 tissue cryosections, stained with antibody against CD31 and biotinylated lectin following interaction with Alexa Fluor streptavidin conjugate. The fraction of perfused vessels was defined as the ratio of lectin positive staining to CD31 positive area fraction (Figure 3A, Figure S3A). Our findings demonstrate that tranilast in combination with doxorubicin or Doxil increased vessel perfusion of 4T1 tumors by 50% and 66%, respectively, whereas chemotherapy or nanomedicine alone had no effect on tumor perfusion compared to the control group (Figure 3C). The relevant improvement in blood flow could be explained by the increase in vessel diameter observed in the tranilast-doxorubicin and tranilast-Doxil treated tumors (Figure 3D). Notably, the total number of vessels remained constant across all treatment groups, suggesting that combinatorial treatments could enhance vascular perfusion without causing pruning of vessels (Figure 3E). These observations were also confirmed in the E0771 tumor model (Figure S4B-C). The combination of the normalization of the tumor vasculature without vessel pruning can rationalize the pronounced reduction by 70% of hypoxia within the microenvironment of tranilast-Doxil treated group (Figure 3B, F). The fractions of hypoxic areas in E0771 primary tumors were measured by pimonidazole injection and staining. Notably, these results are in agreement with previous studies indicating that elimination of ECM with either TGF-β or angiotensin II AT1 inhibition, in mammary tumor models could improve effectiveness of small molecule chemotherapeutics by enhancing transvascular and interstitial drug penetration 20, 33. Given our finding that Doxil enhanced the efficacy of tranilast in terms of interstitial space remodeling and reduction of vessel leakiness, we also examined its potential to modulate the tumor hydraulic conductivity and interstitial fluid pressure (IFP). Hydraulic conductivity is defined as the easiness by which interstitial fluid percolates in tumor ECM and depends on ECM composition. For instance, the negative charge of highly desmoplastic tumors, rich in hyaluronan content, associates with increased flow resistance due to water trapping and limited space available for fluid flow 40. Increased interstitial flow resistance, in turn results in accumulation of interstitial fluid and an increase in IFP 41, 42. By measuring hydraulic conductivity *ex-vivo*, as described elsewhere 21, 36, we found that it was significantly enhanced after tranilast, Doxil, tranilast-doxorubicin or tranilast-Doxil treatment in both 4T1 and E0771 tumors (Figure 3G, Figure S3D). However, the tranilast-Doxil treatment exhibited an outstanding increase (6-fold) compared to the control, as expected due to the efficient elimination of ECM constituents (Figure 3G). Subsequently, we measured the IFP using the wick-in-needle technique 8, 38. In line with the hydraulic conductivity findings and the increased pericyte coverage that reduces vessel leakiness, tranilast-Doxil combination significantly reduced (2-fold) IFP compared to the other groups (Figure 3H, Figure S4E). We further investigated the effect of combinatorial treatments on tumor solid stress by using our previously established tumor opening technique to quantify growth-induced, solid stress in tumors 8, 38 (Figure S6). The data collected indicated that control, doxorubicin and Doxil-treated tumors had larger tumor openings compared to all three tranilast-treated groups, which is a result of lower growth-induced solid stress accumulation in the tranilast-treated tumors (Figure 3I, Figure S4F). Importantly, the accumulation of growth-induced stresses was significantly lower in the tranilast-Doxil group, which exhibited the lowest tumor opening value. We then employed *ex-vivo* stress-strain experiments to determine tumor elasticity and we observed a softening of all tranilast-treated tumors (Figure 3J, Figure S4G-H). Moreover, we assessed the effect on CAFs present in TME by immunostaining with the anti-αSMA, which primarily detects CAFs, and found that tranilast-Doxil significantly reduced the αSMA^+^ fraction *in vivo* (Figure S5).

### Tumor normalization in combination with nanomedicine reprograms macrophages by directing their polarization towards M1 phenotype

**Figure 4:**
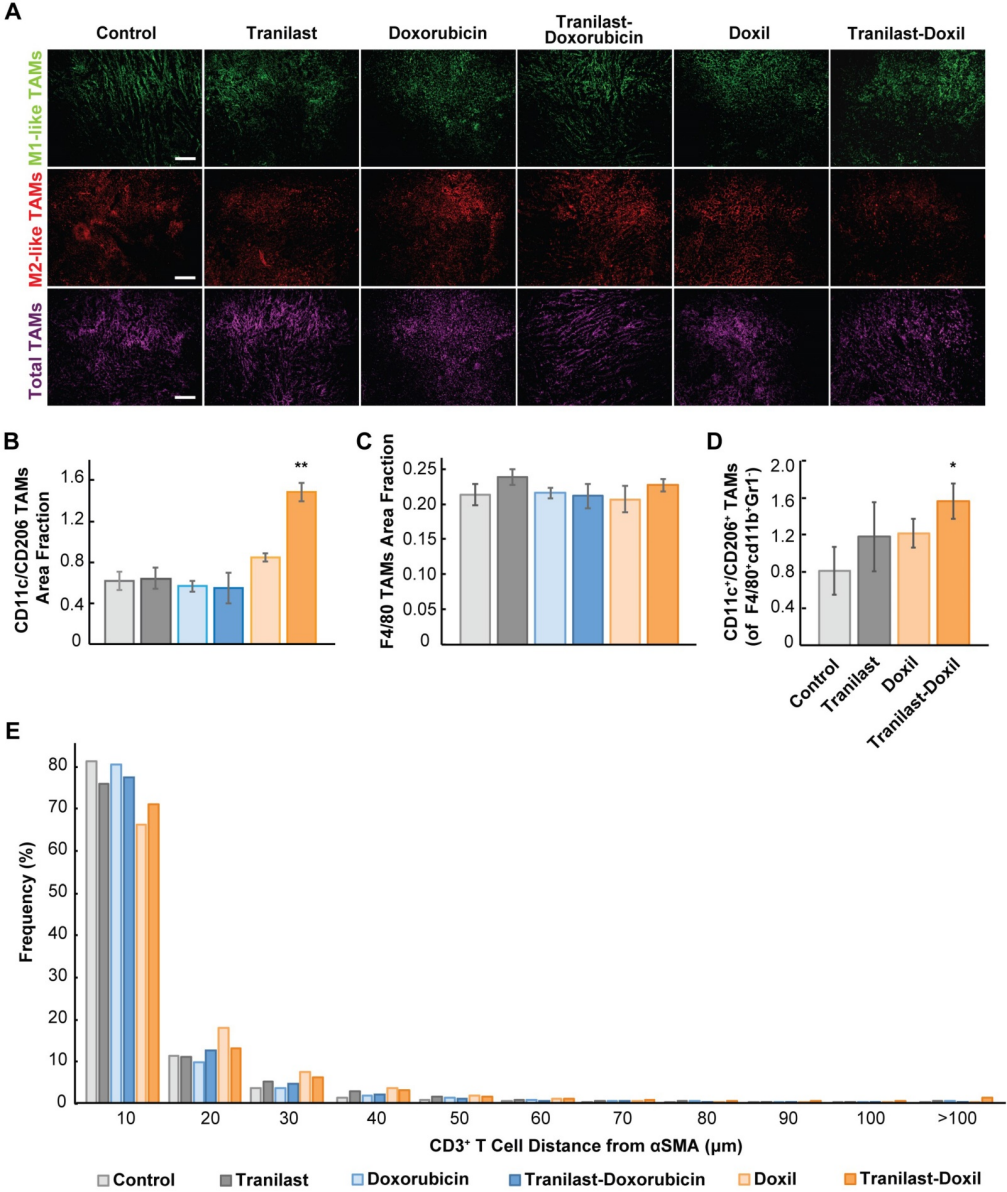
Tumor normalization in combination with nanomedicine reprograms macrophages by directing their polarization towards M1 phenotype. (**A**) Representative images of 4T1 tumor slices immunostained for the M1-like tumor associated macrophage (TAM) marker CD11c (green), the M2-like TAM marker CD206 (red) and the F4/80, which is a pan-macrophage marker (magenta). Quantification of anti-tumoral M1- to M2-like TAM ratio (**B**), and total TAMs area fraction (**C**) in the various treatment groups. (**D**) Ratio of M1-like CD45^+^ CD11b^+^ Gr1^-^ F4/80^+^ CD11c^+^ CD206^-^) to M2-like (CD45^+^ CD11b^+^ Gr1^-^ F4/80^+^ CD11c^-^ CD206^+^) over total TAMs in orthotopic 4T1 breast tumors by flow cytometry analysis. (**E**) Histogram demonstrating distribution of distances between αSMA^+^ CAFs - CD3^+^ T cells. Statistical analyses were performed by comparing the treated groups with the control * and the tranilast-Doxil groups with all other treatment groups **, p≤0.05, (n=8-10). Scale bar: 200μm.

The hypoxic microenvironment triggers myeloid cell infiltration and their differentiation toward tumor-associated macrophages (TAMs)^43^. Importantly, accumulation of TAMs in solid tumors has been correlated with poor therapeutic outcome 26, 44. Considering the positive effects of combinatorial therapy on enhancing oxygen delivery in tumor stroma, we investigated whether it could also modulate the immunosuppressive responses and infiltration of M2-like TAMs. We performed immunofluorescence image analysis in 4T1 and E0771 tumor cryosections followed by staining with antibodies against CD11c and CD206 proteins, which are predominately expressed in M1- and M2-like TAMs, respectively 27, 28. Total macrophage population was calculated from the F4/80 positive fraction (Figure 4A, Figure S7A). We found that only the combination of tranilast with Doxil successfully increased the M1- to M2-like TAMs ratio by more than 2-fold compared to the control group (Figure 4B, Figure S7E). This increase in M1-to M2-like TAM ratio was also confirmed by flow cytometry in which the M1-like TAM population was identified based on the expression of CD45^+^ CD11b^+^ Gr1^-^ F4/80^+^ CD11c^+^ CD206^-^ markers, while the M2-like TAMs based on the expression of CD45^+^ CD11b^+^ Gr1^-^ F4/80^+^ CD11c^-^CD206^+^ (Figure 4D, Figure S9). In addition, taking into consideration that in both tumor models only the tranilast-Doxil combination treatment enhanced the anti-tumor M1-like TAMs and reduced the immunosuppressive M2-like TAM population, while the amount of TAMs remained unaffected between the various treatment groups (Figure 4C, Figure S7, Figure S8B-D), we concluded that combinatorial nanomedicine redirects the M2-like immunosuppressive phenotype towards the M1-like immunosupportive TAMs as a consequence of more functional vasculature and tumor tissue oxygenation.

In addition to TAMs, T cells must also infiltrate the tumor and distribute to cancer cells for effective immune checkpoint blockade (ICB) therapy. Therefore, to further study the TME normalization-driven changes in immune responses, we examined CD3^+^ T cell (CD4^+^ and CD8^+^ T cell) distribution in 4T1 syngeneic tumors possessing a complete immune system after tranilast and doxorubicin or Doxil treatment (Figure S4I-J). T cell infiltration was measured by immunofluorescence image analysis of the density CD3^+^ cells. Doxorubicin and Doxil monotherapies reduced CD3^+^ cell density in tumors, while treatment groups including tranilast had no effect on the density (Figure S4K). Thus, while chemotherapy causes systemic lymphodepletion, increased perfusion in tumors after TME normalization might rescue T cell infiltration.

Once in tumors, T cell migration is restricted by the TME, resulting in decreased efficacy of immunogenic cell death 45. A recent study indicated that a CAFs subpopulation, named CAF-S1, can reduce motility of CD4^+^ and CD45^+^ T lymphocytes via CXCL12 secretion contributing to immunosuppression 31, 46, 47. Additionally, TGF-β 45, 48 and excess collagen 49, which are both reduced by tranilast 36, block T lymphocyte migration in tumors. Thus, we hypothesized that tranilast increases distribution of T cells in tumors. To test this, we measured the distances between CAFs (αSMA positive staining) and CD3^+^ T lymphocytes and plotted the distribution (Figure 4E). We found that TME normalization with tranilast and Doxil increased the fraction of T cells that migrate far (>100 microns) from immunosuppressive CAFs (Figure 4E). This increased migration might result be a result of reduced αSMA density with tranilast treatment. Thus, we further analyzed αSMA-low and -high regions. Indeed, we found that in αSMA-low regions, tranilast increased the distance of CD3^+^ T lymphocytes that migrate from CAFs (Figure S4J). In contrast, in αSMA-high regions, tranilast enabled CD3^+^ T lymphocytes to migrate closer to CAFs (Figure S4J), which suggests that tranilast-treated CAFs could be less suppressive of T cell motility (Figure S4J). This result in αSMA-high regions is in accordance with a recent study of another TGF-β inhibitor in a very desmoplastic breast tumor model 31. The increased T cell distribution with tranilast supports the notion that pre-treatment with tumor normalization agents such as TGF-β inhibitors combined with Doxil might increase the efficacy of immune therapies.

### Enhanced tumor normalization by Doxil nanomedicine improves efficacy of immunotherapy

**Figure 5:**
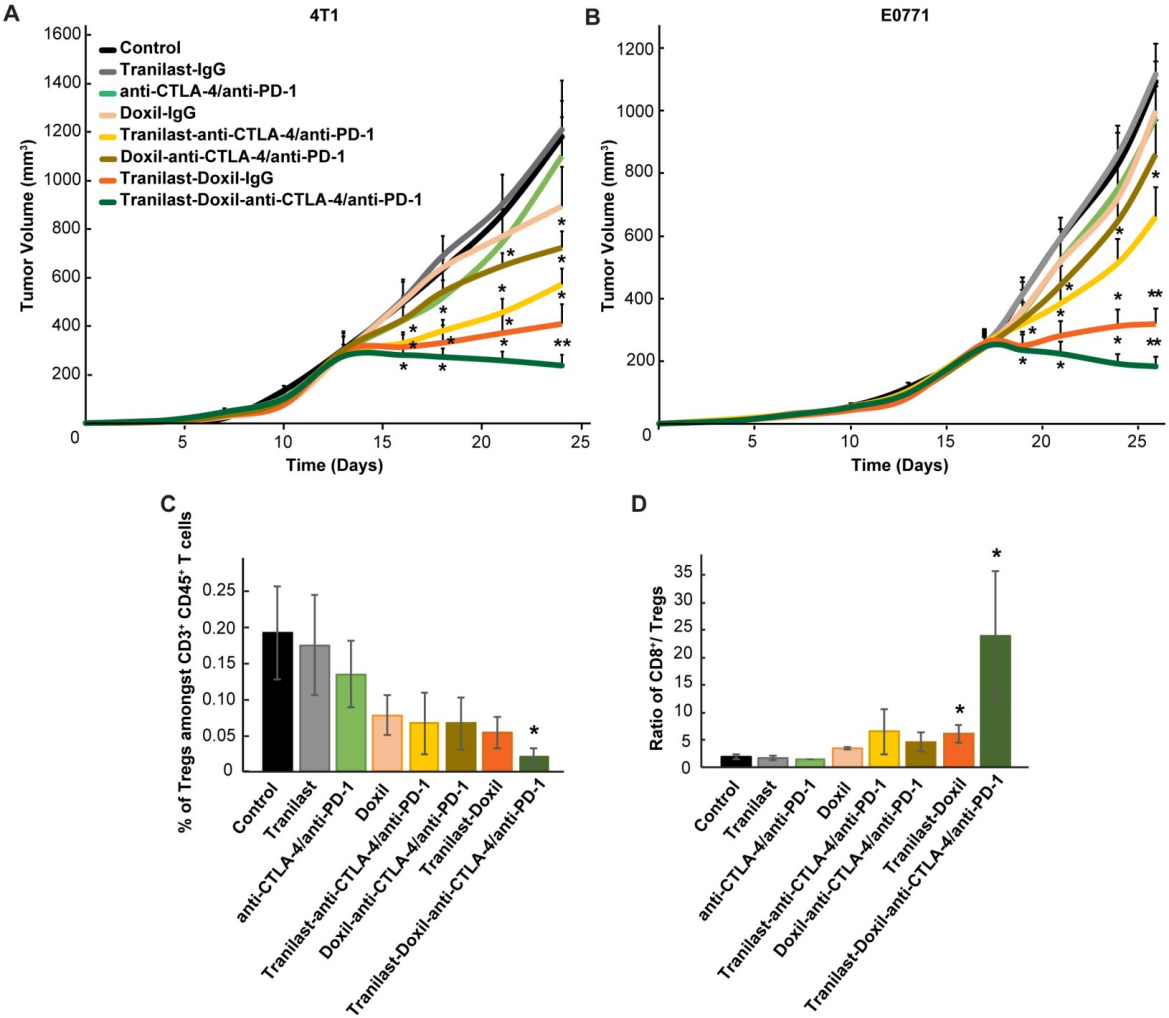
(**A**) Tumor volume curves of Balb/c mice bearing 4T1 tumors and (**B**) C57BL/6 mice bearing E0771 tumors treated with saline (Control), tranilast (200mg/kg), anti-CTLA-4/anti-PD-1 immunotherapy cocktail (5mg/kg and 10mg/kg, respectively), Doxil (3mg/kg), tranilast-anti-CTLA-4/anti-PD-1 immunotherapy cocktail, tranilast-Doxil, Doxil-anti-CTLA-4/anti-PD-1 immunotherapy cocktail and tranilast-Doxil-anti-CTLA-4/anti-PD-1 immunotherapy cocktail. Tranilast-Doxil induced TME normalization in combination with immunotherapy eradicates tumor growth. Statistical analyses were performed by comparing the treated groups with the control * and the tranilast-Doxil- anti-CTLA-4/anti-PD-1 immunotherapy cocktail groups with all other treatment groups **, p≤0.05 (n=8-10). (**C**) Flow cytometry analysis of CD3^+^CD4^+^CD127^lo^CD25^hi^Foxp3^+^ Tregs amongst total CD4^+^ T cells and (**D**) cytotoxic CD8^+^ T cells/Tregs ratio of orthotopic 4T1 breast tumors. Tregs and CD8^+^ levels were assessed using established phenotypic criteria and total CD45^+^CD3^+^ cells were used as common denominator.

To test our hypothesis that improved tumor perfusion, oxygenation and immunostimulation caused by the combined tranilast-Doxil treatment can enhance the efficacy of immunotherapy, we performed a tumor growth study employing a cocktail of immune checkpoint blockers (ICBs). Specifically, we used an immunotherapy cocktail comprising the programmed cell death-1 (PD-1) and cytotoxic T-lymphocyte-associated antigen-4 (CTLA-4) antibodies. Interestingly, immunotherapy cocktail alone did not affect tumor growth while Doxil monotherapy displayed a modest decrease in tumor size (Figure S10B). Combination of immunotherapy with Doxil nanomedicine reduced tumor growth by 40%, while its combination with the tranilast caused a reduction greater than 50% compared to the untreated group. Consistent with our previous results, tranilast-Doxil combinatorial therapy significantly reduced tumor growth by 60%. However, the combination of ICBs with tranilast-Doxil treatment let to the most significant reduction in tumor volume in both mammary tumor models, 4T1 and E0771 (Figure 5A-B, Figure S10). To better understand the effect of immunotherapy on animal survival, we profiled the T cell populations in these tumors by flow cytometry and found that tumors treated with the tranilast-Doxil-ICBs triple combination displayed a significant reduction in intratumoral Foxp3+ Tregs and increased the ratio of cytotoxic CD8^+^ T cell population to Foxp3+ Tregs by 7-fold (Figure 5C-D, Figure S11), all together demonstrating a reversal of immunosuppression. Therefore, our data highlight the use of TME normalization strategies for immunotherapy, in accordance with other recent studies 31, 32 and suggest that TGF-β inhibition could be combined with cytotoxic nanomedicine as a potential therapeutic strategy to improve anti-tumor immunity of highly metastatic and immunotherapy-resistant tumors.

## Conclusion

**Figure 6:**
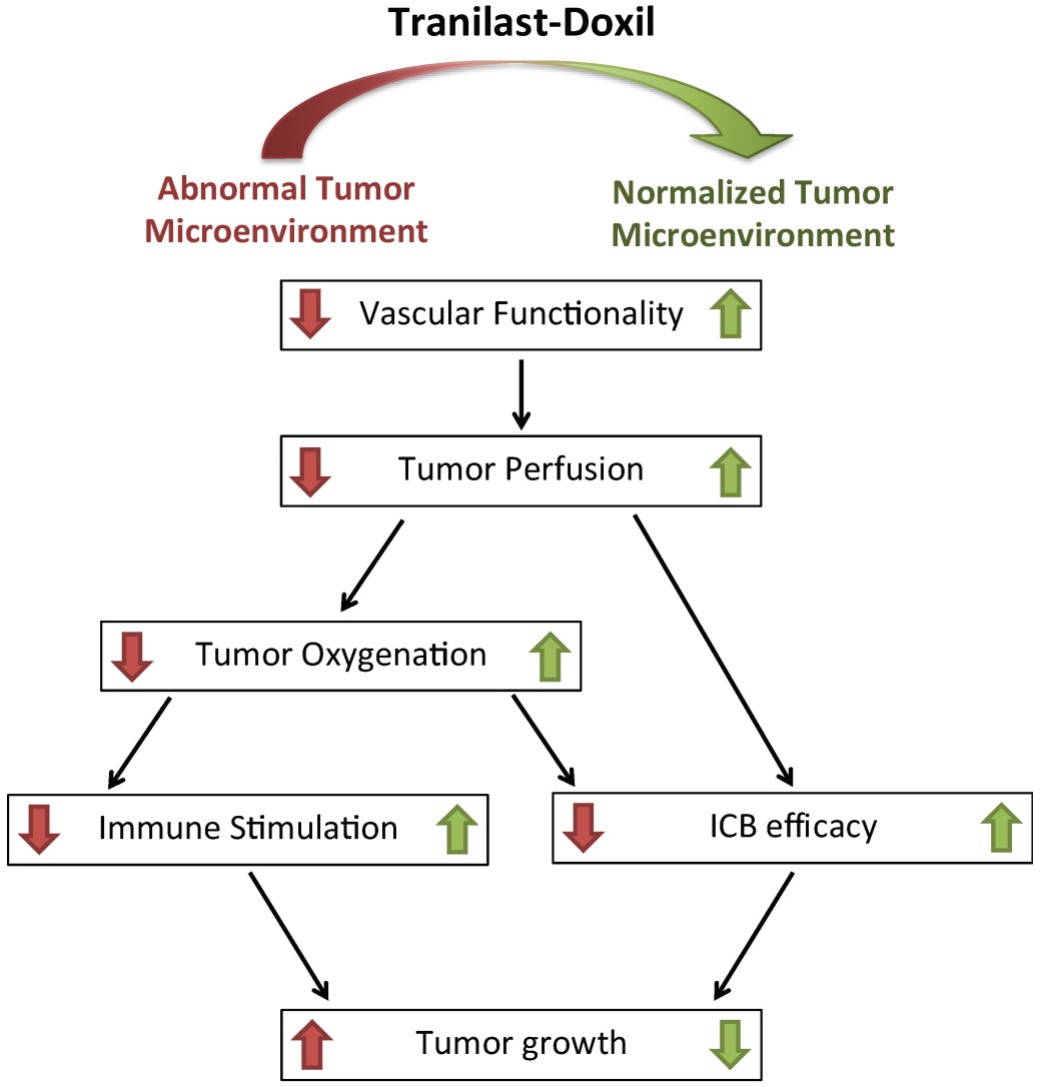
Schematic of the proposed tranilast-induced TME normalization in combination with Doxil nanomedicine mechanism of action. Tranilast-Doxil combinatorial treatment optimizes normalization of the TME by increasing tumor vessel functionality leading to improved perfusion. Improved perfusion results in increased tumor oxygenation and immune stimulation. Improved perfusion and oxygenation enhance the efficacy of ICBs inhibiting primary tumor growth.

Taken together, our data demonstrate that the clinically approved anti-fibrotic and antihistamine drug tranilast in combination with Doxil can effectively prolong overall survival in syngeneic metastatic breast tumor models. Interestingly, the tranilast induced-TME normalization can suppress metastasis when combined with cytotoxic nanomedicine, indicating that the elimination of structural matrix components such as collagen and the subsequent decrease in the intratumoral solid stress impedes cell migration rather than promoting it (Figure S12). This can be attributed to the fact that metastasis is large part due to the lack of oxygenation. By treating hypoxia and improving tumor perfusion and oxygenation, normalization of the TME can inhibit metastasis. We also found that the underlying mechanism to achieve this involves improvement in tumor blood vessel functionality, reprogramming of TAMS and alleviation of hypoxia. Therefore, our study suggests that TME normalization is enhanced when combined with cytotoxic nanomedicine, providing new insights into therapeutic strategies that aim to normalize the TME (Figure 6). Our study also provides a rationale for combining TME normalization agents and nanomedicine with immune therapies, given the enhanced efficacy observed upon the combination of the three. Importantly, there is already clinical evidence that immune therapy added to bevacizumab (Avastin, that can induce vascular normalization) plus chemotherapy regimen improves therapeutic outcome in patients suffering from nonsquamous non-small cell lung cancer, even when liver metastasis is diagnosed 50. Furthermore, a programmed death-ligand 1 (PD-L1) antibody (Tecentriq) combined with nab-paclitaxel chemotherapy (Abraxane) has recently obtained FDA approval as the first immunotherapy drug for PD-L1 positive unresectable or metastatic TNBC.

Our findings demonstrate that both doxorubicin and Doxil monotherapy were more efficient when combined with tranilast (Figure 1). Adding to this, it has been also reported that nanoparticles by themselves can induce normalization effects, which can be additive to the normalization effects of an anti-fibrotic agent 29, 51 or enhance antitumor immune responses 52.

Our findings also identify vessel compression as a main mechanism of resistance to nanoformulations of chemotherapy, which could explain to some extent why nanomedicines have not been successful in drastically increasing overall survival. This is in accordance to our recent findings that metastatic cells can co-opt and eventually compress blood vessels 53. It also provides new insights for the use of nanomedicine and the development of new nanoparticle formulations. We suggest that nanomedicine efficacy can be significantly improved in combination with agents that normalize the TME. Given the fact that the normalization agent (tranilast), the nanomedicine (Doxil) and the immune checkpoint blockers that we employed in our study, are already clinically approved, the findings of our research could be directly translated in clinical trials. As far as the design of new nanoparticle formulations is concerned, current research in nanoparticle development for cancer treatment aims to the design of multifunctional nanoparticle formulations that incorporate several stimuli-responsive or cancer cell targeting features. Increased sophistication, however, most often leads to increased nanoparticle size, which further hinders delivery. Here, we propose the possibility of nanoparticles to carry normalization agents, which will assist even large particles (larger than Doxil, >100nm) to enter and penetrate deeper into the tumor.

Finally, while tranilast was employed as a normalizing agent in this study, other similar agents have also been successfully tested by our group and others (e.g. losartan, pirfenidone, vismodegib, metformin, fasudil, dexamethasone) 20-22, 54-56. In principle, any agent that can induce normalization effects to the TME could be combined with nanomedicine but its degree of efficacy remains to be tested.

## Supplementary Material

Supplementary materials and methods, figures.
