# Supplementary material for: TGF-β inhibition combined with cytotoxic nanomedicine normalizes triple negative breast cancer microenvironment towards anti-tumor immunity
Source: Theranostics. 2020 Jan 12;10(4):1910–22. doi: 10.7150/thno.36936 (PMC6993226; doi:10.7150/thno.36936)
Supplement: Supplementary file 1 — Supplementary materials and methods, figures. [file thnov10p1910s1.pdf]

SUPPORTING INFORMATION

Title: TGF-β inhibition combined with cytotoxic nanomedicine normalizes triple negative breast cancer microenvironment towards anti-tumor immunity

Myrofora Panagi, Chrysovalantis Voutouri, Fotios Mpekris, Panagiotis Papageorgis, Margaret R Martin, John D Martin, Philippos Demetriou, Chryso Pierides, Christiana Polydorou, Andreas Stylianou, Maria Louca, Laura Koumas, Paul Costeas, Kazunori Kataoka, Horacio Cabral, Triantafyllos Stylianopoulos

Materials and Methods

Cell culture. 4T1 and E0771 mouse mammary carcinoma cell lines were purchased from ATCC and CH3 BioSystems, respectively. Both cell lines were maintained in Roswell Park Memorial Institute medium (RPMI, biosera) supplemented with 10% fetal bovine serum FBS and 1% antibiotics.

Drugs and reagents. Tranilast (Rizaben, Kissei Pharmaceutical, Japan) was dissolved in 1% $NaHCO_3$ (33.3 mg/ml) following incubation at 70ºC for an hour, as previously described[1-3]. Doxorubicin hydrochloride (Sigma) was dissolved (625 μg/ml stock) in phosphate buffer saline (PBS). Doxil (Pegylated liposomal doxorubicin, Janssen Pharmaceuticals) was purchased as already made solution (2 mg/ml). The ICBs mouse monoclonal PD-1 (CD279, clone RMP1-14) and mouse monoclonal CTLA-4 (CD152, clone 9D9) were purchased from BioXCell.

Syngeneic tumor models and treatment protocols. We employed two orthotopic syngeneic mammary tumor models, 4T1 and E0771, and six treatment groups; untreated, tranilast, doxorubicin, Doxil, tranilast-doxorubicin and tranilast-Doxil. The untreated-control mice were given saline throughout the study. Tranilast (200 mg/kg) was administered orally once a day from day 4 post-implantation. Previously, we demonstrated that this dose of tranilast successfully induces normalization effects[3]. Also other studies have showed that lower doses of tranilast do not cause any effect on ECM composition[1, 2, 4]. Mice were treated with doxorubicin and Doxil once the tumor volume reached 250mm^3. Doxorubicin (5 mg/kg) was administered via intraperitoneal (i.p.) injection every 72 hours while Doxil (3 mg/kg) via intravenous (i.v.) injection once a week[3, 5-7].

Planar dimensions (x, y) of tumor were monitored every 2-3 days using a digital caliper and tumor volume was estimated from the volume of an ellipsoid and assuming that the third dimension, z, is equal to $\sqrt{x\,y}$. Animal survival was quantified based on the time of death after initiation of treatment or time to reach maximum tumor burden of 1200 mm^3 [8]. All in vivo experiments were conducted in accordance with the animal welfare regulations and guidelines of the Republic of Cyprus and the European Union (European Directive 2010/63/EE and Cyprus Legislation for the protection and welfare of animals, Laws 1994-2013) under a license acquired and approved (No CY/EXP/PR.L14/2019) by the Cyprus Veterinary Services committee, the Cyprus national authority for monitoring animal research for all academic institutions.

Elastic modulus and hydraulic conductivity. Characterization of the mechanical properties and calculation of the elastic modulus were determined using an unconfined compression experimental protocol. Following excision of the primary tumor or the macroscopically metastatic nodules, specimens were loaded on a high precision mechanical testing system (Instron, 5944, Norwood, MA, USA) and compressed to a final strain of 30% with a strain rate of 0.1 mm/min. The dimensions of the primary tumor specimens were 3 × 3 × 2 mm (length × width × thickness), while metastatic nodules were tested as a whole owing to their small size. The elastic modulus was calculated from the slope of the stress-strain curve at the 25-30% strain range[3, 7, 9]. For the calculation of the hydraulic conductivity, stress relaxation experiments were performed in unconfined compression. Specimens underwent four cycles of testing for each of which a 5% compressive strain was applied for 1 minute, followed by a 10 min hold. Subsequently, a common biphasic model of soft tissue mechanics was employed[10], accounting for both the solid phase (cells and extracellular matrix) and the fluid phase (interstitial fluid) of the tumor. The hydraulic conductivity was calculated by fitting the model to the experimental data.

Tumor opening. For the tumor opening measurement, a cut was made along the tumor's longest axis (~80% of its thickness). The tumor was then allowed to relax for 10 min to diminish the effect of resident transient and poroelastic response. The opening at the surface of tumor was then measured using a digital caliper[11, 12].

Interstitial Fluid Pressure. Interstitial fluid pressure (IFP) was measured *in vivo* using the previously described wick-in-needle technique after mice were anesthetized with i.p. injection of Avertin (200mg/kg) and prior to tumor excision[3, 7, 9, 13].

Tumor elasticity using AFM. Primary E0771 tumors were excised and immediately transferred into ice-cold PBS supplemented with a protease inhibitor cocktail (cOmplete Mini, Roce Dianostics GmbH, 1 tablet per 10 mL) [14, 15]. Tumors were then sliced using a 0.5 mm pre-scored tissue cord matrice. Each specimen was immobilized on a plastic dish with a thin layer of two-component fast drying epoxy glue and stored at 4 °C to avoid tissue degradation[14, 15]. AFM measurements were performed 1-72 h post tumor removal, so as to prevent any alterations in stiffness profiles [14, 15]. AFM experiments were carried out on a commercial AFM (Molecular Imaging-Agilent PicoPlus AFM system) was used. Silicon nitride D cantilevers (MLCT Bruker Company with the half-open angle of the pyramidal face of θ ~ 20°, tip radius: 20 nm, frequency in air: 15 kHz) were used in all experiment. The maximum applied loading force was set to 1.8 nN, the exact spring constant k of the cantilever was determined before each experiment using the thermal tune method and the deflection sensitivity was determined in fluid using petri dishes as an infinitely stiff reference material[16]. The collected force curves were

analyzed by AtomicJ [16] so as to calculate the sample's Young's modulus using the Hertz model (the Poisson ratio, v, was set to 0.5).

Fluorescent immunohistochemistry. For immunohistochemistry (IHC) analysis of extracellular matrix (ECM) constituents, 4T1 and E0771 breast tumors were removed, incubated with 4% paraformaldehyde (PFA, Sigma) in PBS for 40 min and washed twice for 10 min with 1xPBS. Fixed tissues were embedded in optimal cutting temperature compound (OCT) in cryomolds (Tissue-Tek) and allowed to freeze completely at -20°C. Transverse 20μm-thick tumor sections of primary tumors and 40μm-thick of lung tissues were produced using the Tissue-Tek Cryo3 (SAKURA). Positively charged HistoBond® microscope slides (Marienfeld) were used to bound four tissue sections per tumor. Tumor sections were then incubated in blocking solution (10% fetal Bovine Serum, 3% Donkey Serum, 1x PBS) for 2 hr and immunostained with the following primary antibodies; rabbit anti-collagen I (ab34710, Abcam 1:100), sheep anti-hyaluronic acid (ab53842, Abcam 1:100), rabbit anti-αSMA (ab5694, Abcam 1:100), rat anti-CD3 (17A2, BioLegend 1:100), hamster anti-CD11c (HL3, BD Pharmingen 1:100), rat anti-CD206 (MR5D3, BIO-RAD 1:50) and rat anti-F4/80 (A3-1, BIO-RAD 1:50), overnight at 4°C. Secondary antibodies against rabbit, sheep, rat or hamster conjugated to Alexa Fluor 488, 555 and 647 (Invitrogen) were used at 1:400 dilution. All samples were incubated in secondary antibody solution including DAPI (Sigma, 1:100 of 1mg/ml stock) for 2 hr at room temperature in the dark. Sections were mounted on microscope slides using the ProLong™ Gold Antifade Mountant (Invitrogen) and covered with glass coverslip.

Collagen I and Hyaluronan. Tissue cryosections of 4T1 and E0771 primary breast tumors and lungs were immunostained with anti-Collagen I (ab34710, Abcam 1:100) and anti-hyaluronic acid (ab53842, Abcam 1:100) and signal was detected with Alexa Fluor-647 anti-rabbit IgG (H+L) (A21244, Invitrogen 1:400) and Alexa Fluor-488 anti-sheep IgG (H+L) (A11015, Invitrogen 1:400) secondary antibodies, respectively.

Vascular perfusion. To assess functional vasculature, mice were anesthetized with with i.p. injection of Avertin (200mg/kg) and slowly injected intracardially with 100 μl (4mg/kg) of biotinylated lycopersicon esculentum lectin (B-1175, Vector Labs) which was allowed to distribute throughout the body for 7 min[8]. Finally, mice were sacrificed via CO_2 inhalation and tumors/lungs were removed. Excised tumors/lungs were fixed in 4% PFA (Sigma) and processed as described above for IHC analysis. The number of blood vessels was measured from the positive staining of the endothelial marker CD31 (MEC13.3, BD Pharmingen 1:100) while the area fraction of perfused vessels was determined as the ratio of lectin and CD31 overlapping staining to CD31 positive staining. CD31 signal was detected with Alexa Fluor-647 Goat Anti-Rat IgG (H+L) (Invitrogen, A-21247, 1:400) secondary antibody and lectin staining with Streptavidin Alexa Fluor 488 conjugate (Invitrogen, S11223, 1:200 dilution).

Pericyte coverage of perfused blood vessels. For pericyte coverage analysis, tisuue cryosections of 4T1 and E0771 primary tumors were immunostained with primary anti-CD31 antibody (MEC13.3, BD Pharmingen 1:100) and the anti-αSMA antibody (ab5694, Abcam 1:100), while vascular perfusion was detected as described above. CD31 signal was detected with Alexa Fluor-647 anti-rat IgG (H+L) (A21247, Invitrogen 1:400) and αSMA signal with Alexa Fluor-555 anti-rabbit IgG (H+L) (A21428, Invitrogen 1:400) secondary antibodies, while lectin staining was visualized with Streptavidin Alexa Fluor-488 conjugate (Invitrogen, S11223, 1:200 dilution) as previously described. Pericyte coverage of functional vessels was determined as the ratio of lectin, αSMA and CD31 positive staining to total CD31 area fraction.

Hypoxia studies. Mice bearing orthotopic E0771 breast tumors were injected with 60mg/kg of pimonidazole HCl at 60mg/kg 2 hr prior to tumor removal[8]. Primary tumors were then excised, fixed in 4% PFA, embedded in OCT and processed accordingly for IHC. Hypoxic regions were detected using the mouse anti-pimonidazole RED 549 conjugate antibody (HP7-100Kit, 1:100). Hypoxic area fraction across different treatment groups was normalized to DAPI staining.

T cell-CAF distance. Distances between $CD3^+$ T cells and the corresponding nearest αSMA^+ area were calculated using custom MATLAB scripts with built-in image processing functions. $CD3^+$ T cells were determined automatically based on signal intensity, size, and morphology thresholds and αSMA^+ area was similarly determined based on an intensity threshold. The thresholds were chosen manually by reviewing the attributes of all images, and the same thresholds were used for all images. Then, the centroids of $CD3^+$ T cells were determined, and each cell's distance to the nearest αSMA^+ area was measured by finding the distance between the centroids and the nearest αSMA^+ pixels. Also, images were divided into two groups. One group contains images that represent αSMA-rich tissue and the other group contains images that are αSMA-poor. The average αSMA^+ area fraction was calculated and used as the parameter to determine which images displayed a high or low αSMA^+ area fraction. Quantification of αSMA positive staining was normalized to the total image area among different treatment groups.

Determination of macrophage content in primary tumors. Macrophage status of 4T1 and E0771 primary tumor microenvironment and E0771 lung macrometastatic nodules was determined following immunostaining with anti-CD11c (HL3, BD Pharmingen 1:100), rat anti-CD206 (MR5D3, BIO-RAD 1:50) and rat anti-F4/80 (A3-1, BIO-RAD 1:50) antibodies to detect M1-like TAMs, M2-like TAMs and total TAM population, respectively. M1-like TAM signal was visualized with Alexa Fluor-488 anti-hamster IgG (H+L) (ab173003, Abcam 1:200) secondary antibody while M2-like and total TAMs were detected with Alexa Fluor-647 anti-rat IgG (H+L) (A21247, Invitrogen 1:400) secondary antibody. Quantification of M1-like TAMs was defined as the ratio of CD11c and F4/80 overlapping signal to F4/80 positive signal. M2-like TAM content was indicated by CD206 area fraction after subtraction of the CD11c and CD206 overlapping signal. All measurements were normalized to DAPI positive staining.

Image acquisition. Images of immunelabeled tumor sections from the interior and periphery were acquired at 10x magnification using the Olympus BX53 fluorescence microscope. In order for the images to be comparable they were taken at the same settings and analyzed using a previously developed in-house code in MATLAB (MathWorks, Inc., Natick, MA, USA)[3, 7, 9, 11].

Doxil-induced DNA damage. Mice were orthotopically implanted with 4T1 mouse mammary cancer cells as described earlier and divided into four groups. The first group was orally treated with tranilast (200 mg/kg) once a day from day 4 post-implantation and the second group was given an equal volume of saline until completion of treatment. The third group of mice received one dose of Doxil (3 mg/kg) via intravenous (i.v.) injection while the forth group received one dose of Doxil (3mg/kg) in combination with tranilast (200 mg/kg) once tumor volume reached 200 mm^3. Tumors were harvested and fixed in 4% PFA, dehydrated through a series of graded ethanol washes and embedded in paraffin. Tranverse 7μm-thick tissue sections were produced using the microtome (Accu-Cut SRM 200 Rotary Microtome, SAKURA), flatten out into water and allowed to dry overnight at 37°C. Sections were then deparaffinized and subjected to antigen retrieval (microwave heat treatment with TriSodium Citrate, pH 6, for 20 min). Tissue sections were then washed with 1x PBS and incubated in blocking serum (10% Donkey serum, 3% Fetal Bovine Serum, 1x PBS) for 2 hr at room temperature. Next, tissue sections were immunostained with the primary anti-γH2A.X antibody (ab22551, Abcam 1:100) and incubated overnight at 4°C. The antibody-antigen interaction was detected using the Mouse Polymer IHC Kit (ab127055, Abcam) according to the manufacture's instructions and finally visualized by adding a DAP chromogen (ab94665, Abcam) that yields a permanent brown precipitate . Following immunostaining, slides were counterstained with hematoxyline, rehydrated and mounted with DPX mountant for histology (Sigma).

Supporting Figures

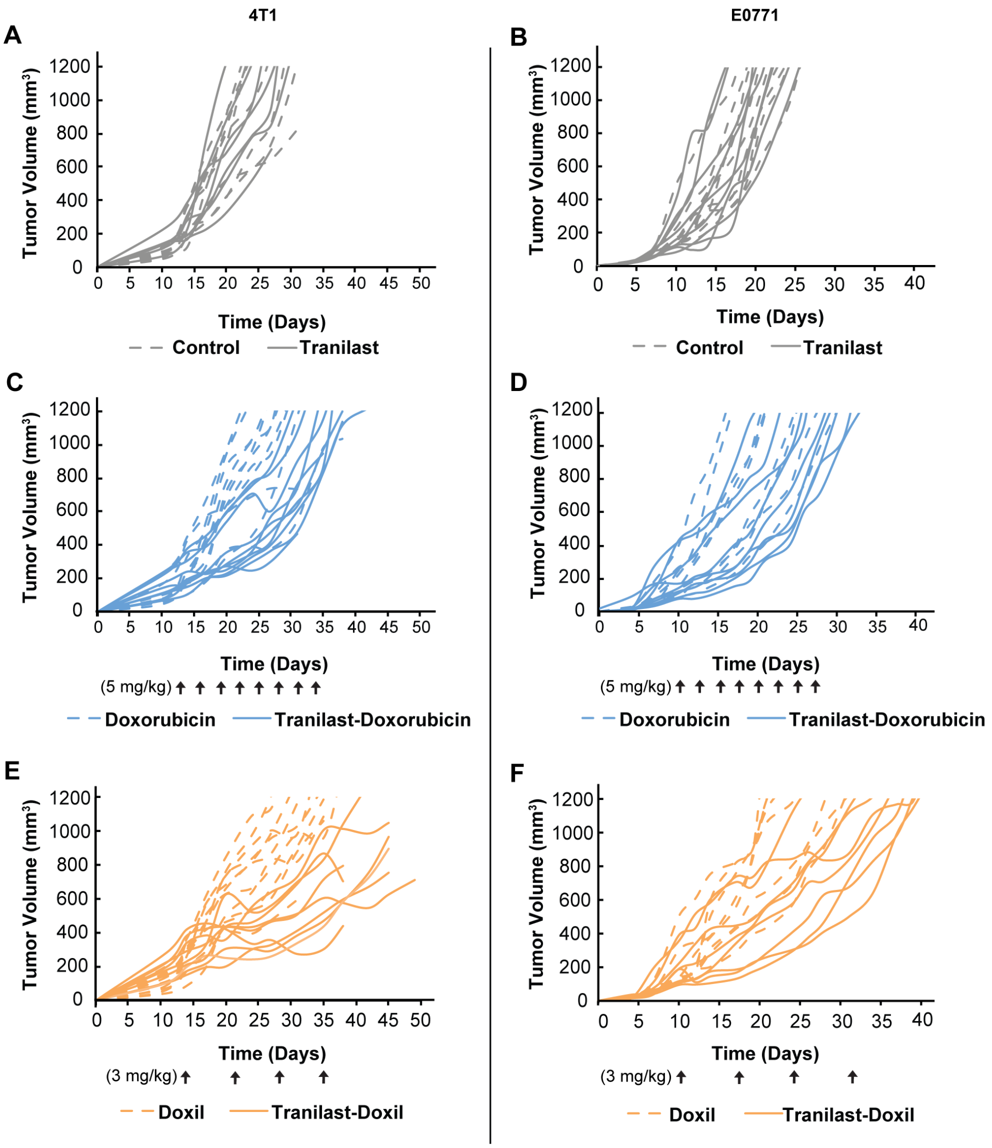

Figure S1. Combination of tranilast with Doxil nanomedicine significantly inhibits tumor growth. Individual growth curves of syngeneic orthotopic 4T1 and E0771 murine breast tumors implanted in female BALB/c and C57BL/6 mice, respectively. Tranilast-treated mice displayed similar tumor volumes in both 4T1 (**A**) and E0771 (**B**) tumor models, as compared to the control group. Tranilast (200mg/kg) was administered daily using oral gavage from day 4 post-implantation until time of death or time to reach a tumor burden of 1200 mm^3. Combination of tranilast with doxorubicin chemotherapy had a moderate effect on tumor growth when compared to doxorubicin monotherapy. (**C-D**) Doxorubicin (5mg/kg) was administered via intraperitoneal (i.p.) injection from day 13 post-implantation of 4T1 cells and from day 10 post-implantation of E0771 cells. Doxorubicin injection was repeated every 72 hr. Tranilast-Doxil combinatorial therapy induced a strong tumor growth inhibition versus Doxil monotherapy. (**E-F**) Doxil (3mg/kg) was intravenously injected on days 14, 21, 28 and 35 post-implantation of 4T1 cells

and days 10, 17, 24 and 31 post-implantation of E0771 cells. Statistical analyses were performed by comparing the treated groups with the control * and the tranilast-Doxil group with all other treatment groups **. A *P value* of less than or equal to 0.05 was considered statistically significant.

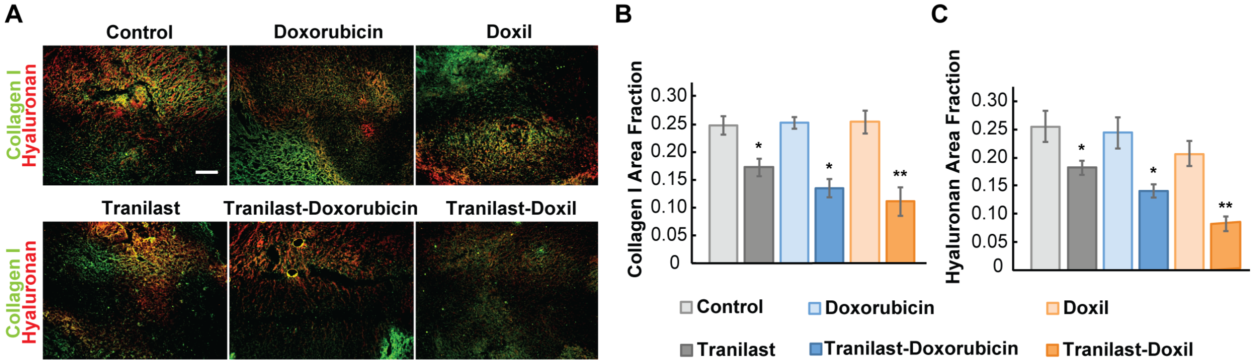

Figure S2. Tranilast-Doxil normalizes E0771 tumor microenvironment by decreasing ECM components. (**A**) Representative fluorescence images of Collagen I (green) and Hyaluronan (red) immunostaining of E0771 breast tumors treated as indicated. Quantification of Collagen I (**B**) and Hyaluronan (**C**) area fractions. Statistical analyses were performed by comparing the treated groups with the control * and the tranilast-Doxil group with all other treatment groups **, p≤0.05, (n=8-10). Scale bar: 200μm.

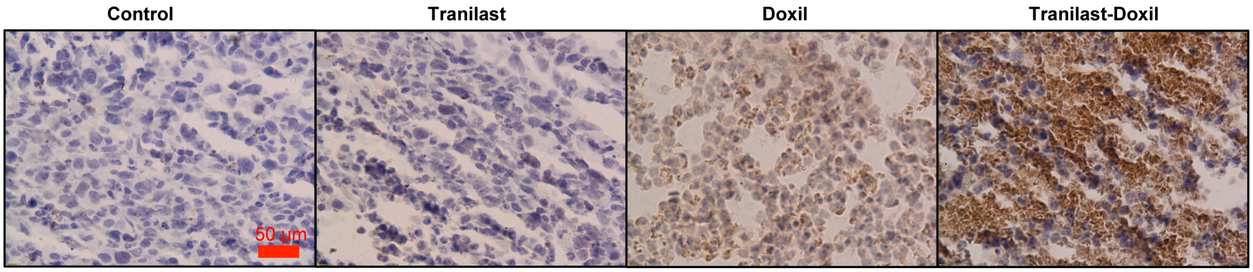

Figure S3. Representative images of DNA damage (brown color) by γH2A.X immunostaining of paraffin fixed tissue sections of 4T1 tumor bearing mice following treatment with saline (control), tranilast (200mg/kg), Doxil (3mg/kg) and tranilast-Doxil. Images demonstrate increased DMA damage for the Tranilast-Doxil group indicating that tranilast-induced TME normalization strategy enhances delivery of Doxil nanomedicine to the tumor. Doxil-induced DNA damage results to double strand DNA breaks shown with brown precipitate. Cell nuclei were stained with hematoxylin (blue color). Scale bar: 50μm.

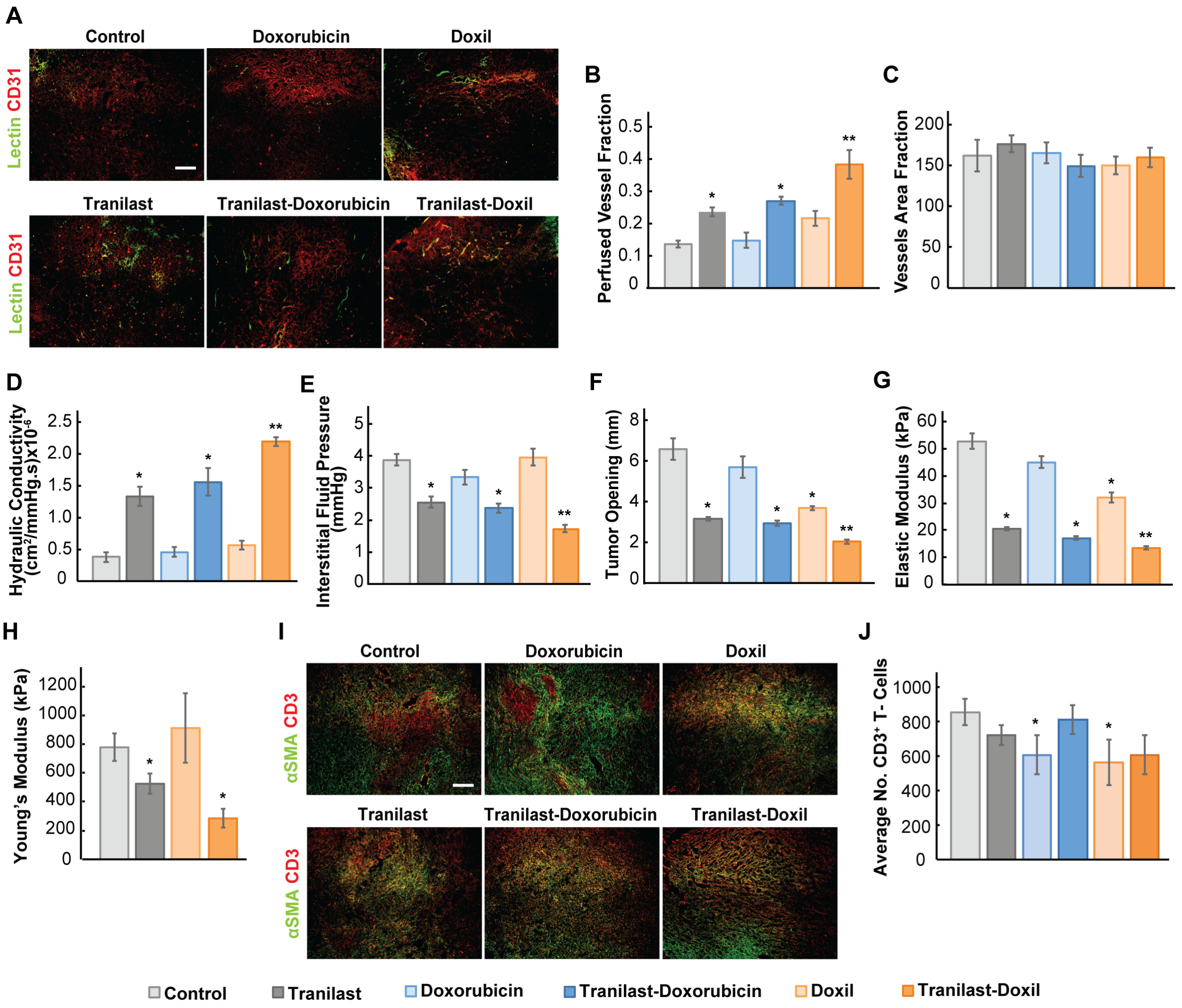

K

αSMA^HIGH			αSMA^LOW		
	αSMA-CD3 Mean Distance	±		αSMA-CD3 Mean Distance	±
**Control**	6.02	1.61	**Control**	10.69	1.63
**Tranilast**	3.87	1.15	**Tranilast**	22.03	6.08
**Doxorubicin**	8.67	2.95	**Doxorubicin**	7.79	–
**Tranilast-Doxorubicin**	9.18	2.47	**Tranilast-Doxorubicin**	12.43	1.20
**Doxil**	18.87	12.21	**Doxil**	16.87	2.23
**Tranilast-Doxil**	8.41	1.46	**Tranilast-Doxil**	17.10	3.52

Figure S4. Tranilast-Doxil combination improves perfusion by alleviating intratumoral fluid and solid pressure and increases T cell infiltration. (**A**) Representative fluorescence images of E0771 breast tumor slices immunostained for biotynilated tomato lectin (green) and CD31 (red) after various treatments as indicated. (**B**) Quantification of CD31 and lectin co-expression indicating vascular perfusion. (**C**) Quantification of CD31 (red) positive staining indicating total blood vessel fraction. (**D**) Quantification of hydraulic conductivity and (**E**) interstitial fluid pressure measurements of E0771 tumors treated as indicated. (**F**) Sample-blind measurements of tumor opening and (**G**) elastic modulus measurements following excision of E0771 tumors. (**H**)

Nanoscale elastic modulus measurements following excision of E0771 tumors using AFM. (**I**) Representative fluorescence images of 4T1 breast tumor slices immunostained for CD3 T cell marker (red) and αSMA (green). (**J**) Quantification of $CD3^+$ T cell density. (**K**) Average distance of $CD3^+$ T cells from αSMA^+ CAFs upon different treatment in αSMA-rich and -poor tumor tissue. Statistical analyses were performed by comparing the treated groups with the control * and the tranilast-Doxil group with all other treatment groups **, p≤0.05, (n=8-10). Scale bar: 200μm.

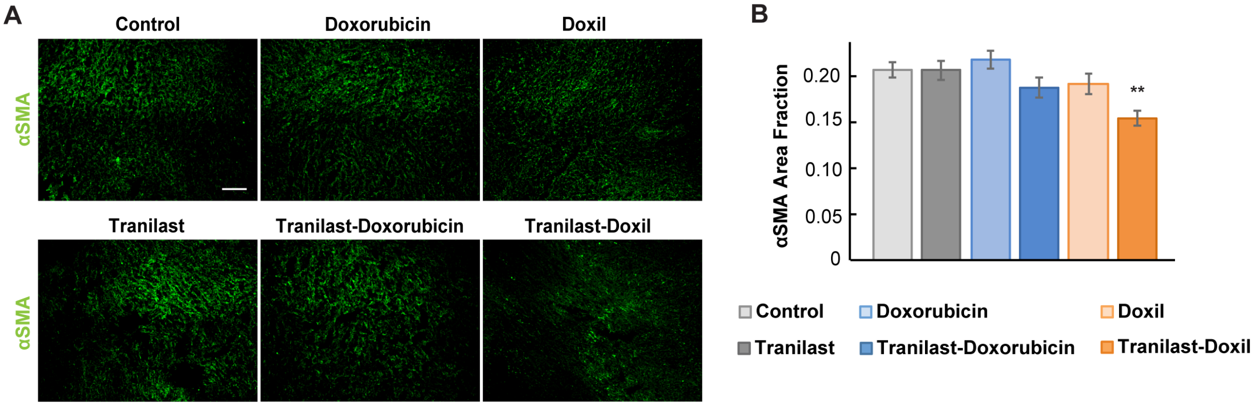

Figure S5. (**A**) Representative fluorescence images of 4T1 breast tumors immunostained with the αSMA protein marker (primarily CAF) treated as indicated. (**B**) Quantification of αSMA area fraction. Statistical analyses were performed by comparing the treated groups with the control * and the tranilast-Doxil group with all other treatment groups **, p≤0.05, (n=8-10). Scale bar: 200μm.

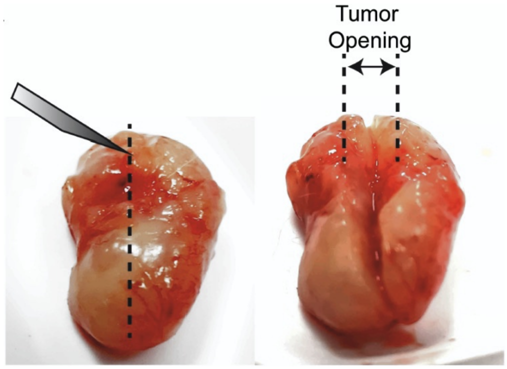

Figure S6. Description of the tumor opening experiment. Following tumor excision, a cut is made to the tumor along its long axis, approximately 80% of the tumor thickness (left). The tumor deforms as a result of the release of the growth-induced, residual stresses and the tissue relaxes in a measurable way. To quantify the degree of relaxation, the tumor opening is measured. (Images reproduced with permission from Ref. [12]).

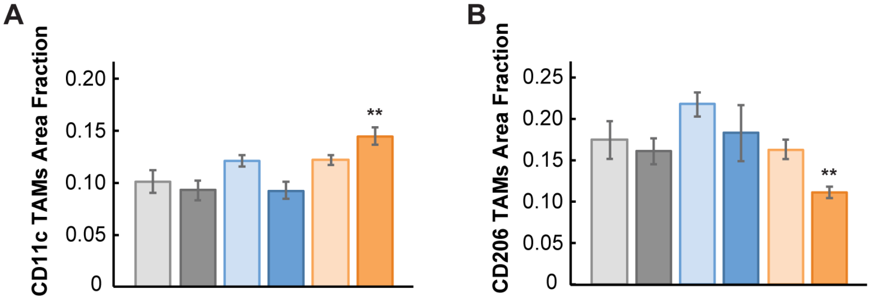

Figure S7. Quantification of anti-tumoral (**A**) M1- and (**B**) M2-like TAM area fraction in 4T1 tumors in the various treatment groups. Statistical analyses were performed by comparing the treated groups with the control * and the tranilast-Doxil group with all other treatment groups **, p≤0.05.

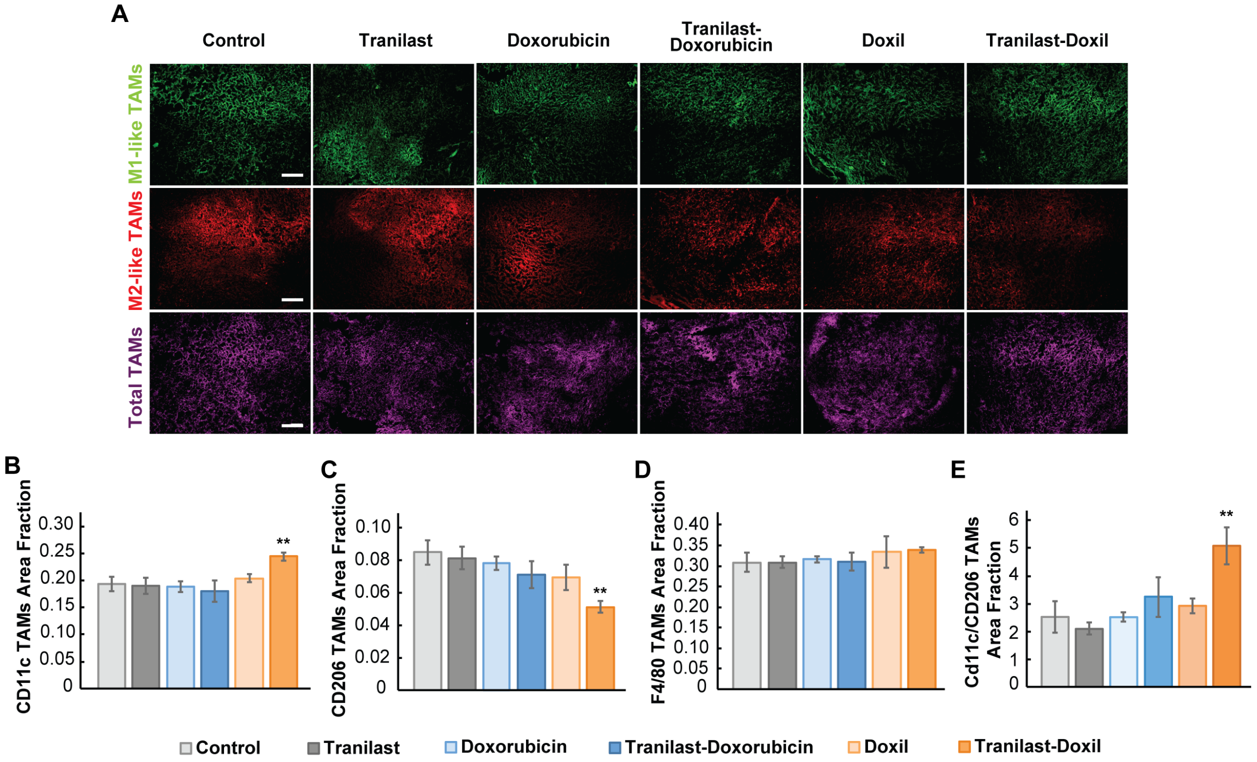

Figure S8. Tranilast-Doxil combinatorial treatment increases the antitumor immune responses of E0771 primary tumors by directing macrophages polarization towards M1 phenotype. (**A**) Representative images of E0771 tumor slices immunostained for the M1-like tumor associated macrophage (TAM) maker CD11c (green), the M2-like TAM marker CD206 (red) and the F4/80, which is a pan-macrophage marker (magenta). Quantification of anti-tumoral (**B**) M1- and (**C**) M2-like and (**D**) total TAMs area fraction in various treatment groups. (**E**) Ratio of M1- to M2-like TAM ratio. Statistical analyses were performed by comparing the treated groups with the control * and the tranilast-Doxil group with all other treatment groups **, p≤0.05, (n=8-10). Scale bar: 200μm.

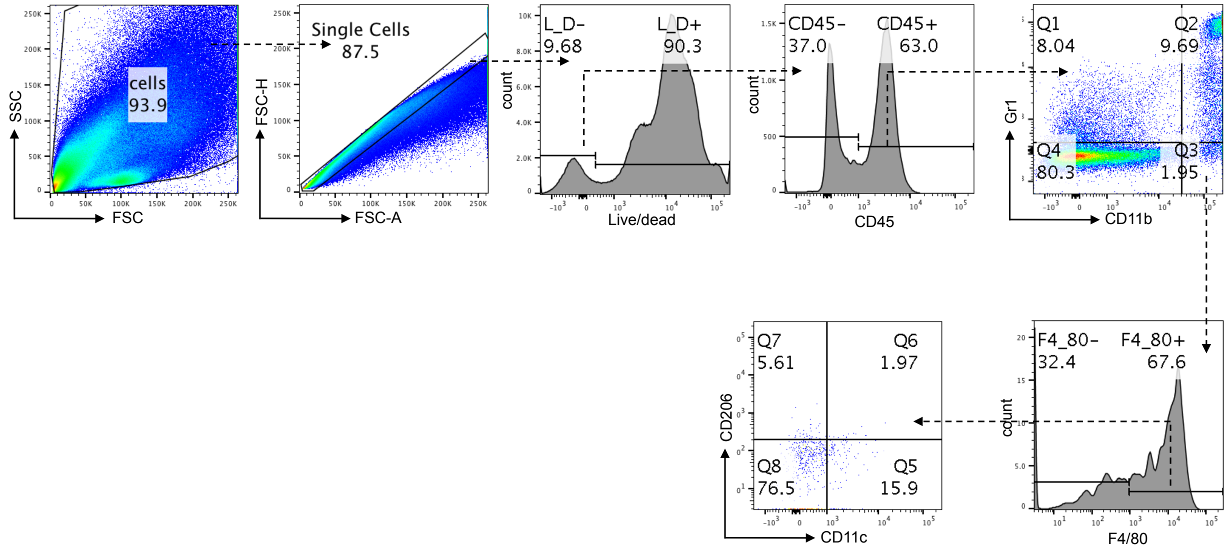

Figure S9. Example analysis of TAM phenotypes in 4T1 tumor model. Cells were gated on FSC/SSC parameters. TAMs were then analyzed based on their expression of both CD11b and absence of Gr1 cell surface markers. F4/80 TAMs were gated from the CD11b^{+} Gr1^{-} gate, which then was subdivided into M2-like TAMs based on the expression of CD206 marker and M1-like TAMs based on the expression of CD11c surface marker. Values noted are percentages of the parent gate

A

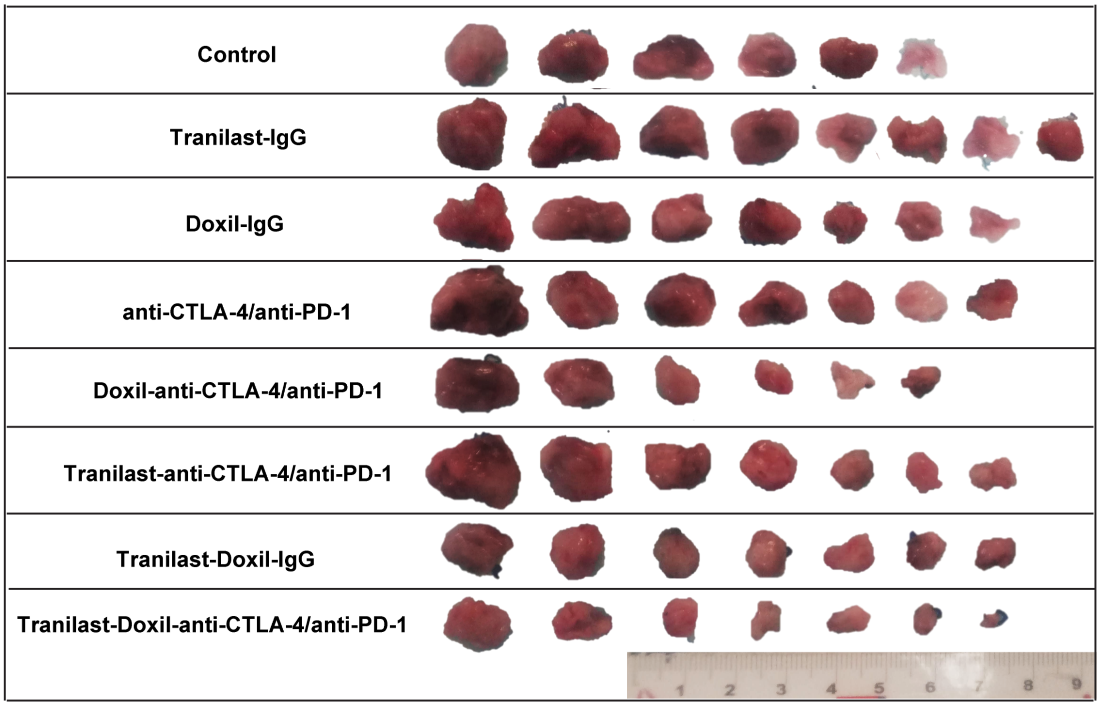

B

CTLA4/anti-PD-1 immunotherapy Vs tranilast-Doxil-anti-CTLA4/anti-PD-1 immunotherapy			
4T1		E0771	
Time point	*p-value*	Time point	*p-value*
Day 16	0.055	Day 19	0.1331
Day 18	0.0092	Day 21	0.0121
Day21	0.0003	Day24	0.0005
Day 24	9.8E-05	Day 26	9,.4E05

C

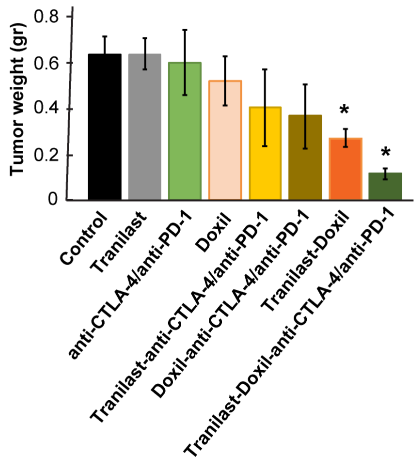

Figure S10. (**A**) Representative images of E0771 primary tumors treated as indicated. (**B**) Comparison between anti-CTLA4/anti-PD-1 immunotherapy and tranilast-Doxil-anti-CTLA4/anti-PD-1 immunotherapy, we performed Paired Student's t-test ($p \leq 0,05$) adjusted by the Bonferroni correction ($p \leq 0,025$). (**C**) E0771 tumor weight quantification. Statistical analyses were performed by comparing the treated groups with the control, * denotes statistically significant difference ($p \leq 0.05$).

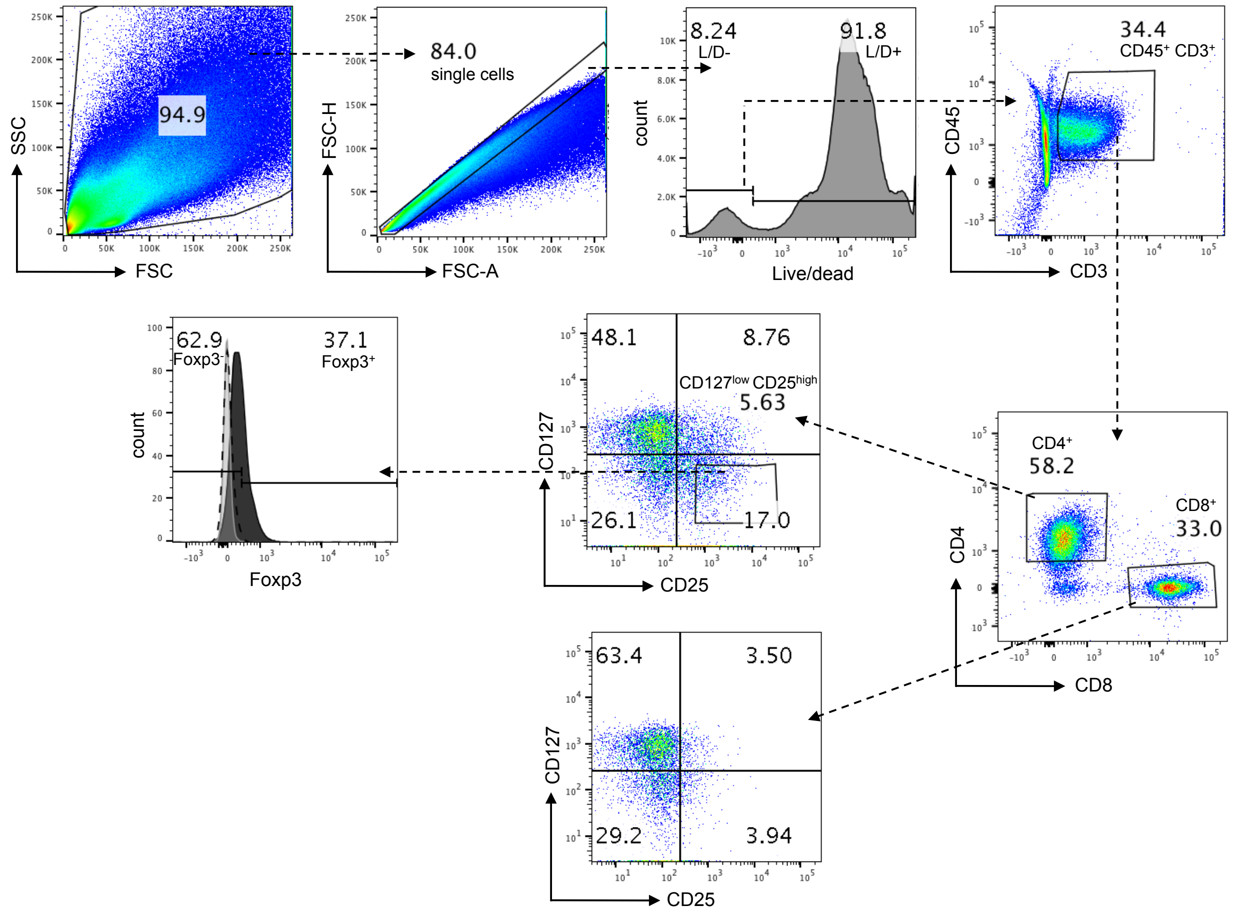

Figure S11. Example analysis of T cell phenotypes in 4T1 tumor model. Lymphocytes were gated on FSC/SSC parameters. T cells were analyzed based on their expression of both CD45 and CD3 and then separated into $CD4^+$ or $CD8^+$. Regulatory T cells (Tregs) were identified based on the high expression of CD25 cell surface marker, low expression of CD127 and high expression of the Foxp3 transcription marker. Values noted are percentages of the parent gate.

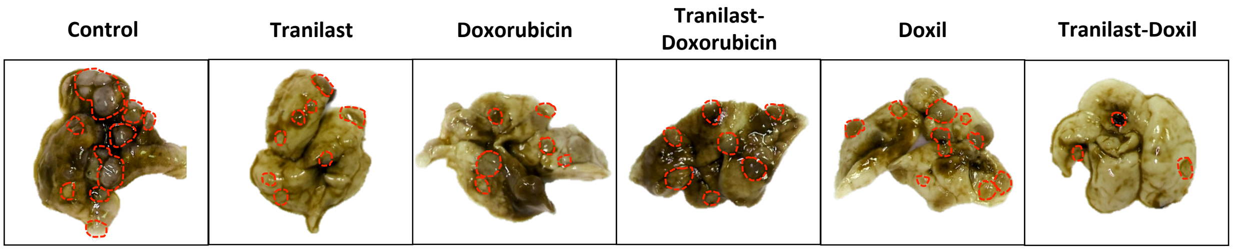

Figure S12. Representative lung photographs of 4T1 murine breast tumors treated as indicated. Formation of lung macrometastases is dramatically reduced in tranilast-Doxil treated mice.

REFERENCES

1. Hiroi M, Onda M, Uchida E, Aimoto T. Anti-tumor effect of N-[3,4-dimethoxycinnamoyl]-anthranilic acid (tranilast) on experimental pancreatic cancer. J Nippon Med Sch. 2002; 69: 224-34.
2. Chakrabarti R, Subramaniam V, Abdalla S, Jothy S, Prud'homme GJ. Tranilast inhibits the growth and metastasis of mammary carcinoma. Anticancer Drugs. 2009; 20: 334-45.
3. Papageorgis P, Polydorou C, Mpekris F, Voutouri C, Agathokleous E, Kapnissi-Christodoulou CP, et al. Tranilast-induced stress alleviation in solid tumors improves the efficacy of chemo- and nanotherapeutics in a size-independent manner. Sci Rep. 2017; 7: 46140.
4. Platten M, Wick W, Wischhusen J, Weller M. N-[3,4-dimethoxycinnamoyl]-anthranilic acid (tranilast) suppresses microglial inducible nitric oxide synthase (iNOS) expression and activity induced by interferon-gamma (IFN-gamma). Br J Pharmacol. 2001; 134: 1279-84.
5. Diop-Frimpong B, Chauhan VP, Krane S, Boucher Y, Jain RK. Losartan inhibits collagen I synthesis and improves the distribution and efficacy of nanotherapeutics in tumors. Proc Natl Acad Sci U S A. 2011; 108: 2909-14.
6. Chauhan VP, Stylianopoulos T, Martin JD, Popovic Z, Chen O, Kamoun WS, et al. Normalization of tumour blood vessels improves the delivery of nanomedicines in a size-dependent manner. Nature Nanotechnol. 2012; 7: 383-8.
7. Mpekris F, Papageorgis P, Polydorou C, Voutouri C, Kalli M, Pirentis AP, et al. Sonic-hedgehog pathway inhibition normalizes desmoplastic tumor microenvironment to improve chemo- and nanotherapy. J Control Release. 2017; 261: 105-12.
8. Chauhan VP, Martin JD, Liu H, Lacorre DA, Jain SR, Kozin SV, et al. Angiotensin inhibition enhances drug delivery and potentiates chemotherapy by decompressing tumor blood vessels. Nat Commun. 2013; 4: 10.1038/ncomms.3516.
9. Polydorou C, Mpekris F, Papageorgis P, Voutouri C, Stylianopoulos T. Pirfenidone normalizes the tumor microenvironment to improve chemotherapy. Oncotarget. 2017; 8: 24506-17.
10. Angeli S, Stylianopoulos T. Biphasic modeling of brain tumor biomechanics and response to radiation treatment. J Biomech. 2016; 49: 1524-31.
11. Stylianopoulos T, Martin JD, Chauhan VP, Jain SR, Diop-Frimpong B, Bardeesy N, et al. Causes, consequences, and remedies for growth-induced solid stress in murine and human tumors. Proc Natl Acad Sci U S A.. 2012; 109: 15101-8.
12. Voutouri C, Stylianopoulos T. Accumulation of mechanical forces in tumors is related to hyaluronan content and tissue stiffness. PloS one. 2018; 13: e0193801.
13. Fadnes HO, Reed RK, Aukland K. Interstitial fluid pressure in rats measured with a modified wick technique. Microvasc Res. 1977; 14: 27-36.
14. Plodinec M, Loparic M, Monnier CA, Obermann EC, Zanetti-Dallenbach R, Oertle P, et al. The nanomechanical signature of breast cancer. Nat Nanotechnol. 2012; 7: 757-65.
15. Tian M, Li Y, Liu W, Jin L, Jiang X, Wang X, et al. The nanomechanical signature of liver cancer tissues and its molecular origin. Nanoscale. 2015; 7: 12998-3010.
16. Stylianou A, Gkretsi V, Patrickios CS, Stylianopoulos T. Exploring the Nano-Surface of Collagenous and Other Fibrotic Tissues with AFM. Methods Mol Biol. 2017; 1627: 453-89.
